# Respiratory supercomplexes enhance electron transport by decreasing cytochrome *c* diffusion distance

**DOI:** 10.15252/embr.202051015

**Published:** 2020-10-05

**Authors:** Jens Berndtsson, Andreas Kohler, Sorbhi Rathore, Lorena Marin‐Buera, Hannah Dawitz, Jutta Diessl, Verena Kohler, Antoni Barrientos, Sabrina Büttner, Flavia Fontanesi, Martin Ott

**Affiliations:** ^1^ Department of Biochemistry and Biophysics Stockholm University Stockholm Sweden; ^2^ Department of Molecular Biosciences The Wenner‐Gren Institute Stockholm University Stockholm Sweden; ^3^ Department of Neurology Miller School of Medicine University of Miami Miami FL USA; ^4^ Department of Biochemistry and Molecular Biology Miller School of Medicine University of Miami Miami FL USA; ^5^ Institute of Molecular Biosciences University of Graz Graz Austria; ^6^ Department of Medical Biochemistry and Cell Biology University of Gothenburg Gothenburg Sweden

**Keywords:** bioenergetics, competitive fitness, cryo‐EM, mitochondria, respiratory chain supercomplexes, Metabolism, Structural Biology, Organelles

## Abstract

Respiratory chains are crucial for cellular energy conversion and consist of multi‐subunit complexes that can assemble into supercomplexes. These structures have been intensively characterized in various organisms, but their physiological roles remain unclear. Here, we elucidate their function by leveraging a high‐resolution structural model of yeast respiratory supercomplexes that allowed us to inhibit supercomplex formation by mutation of key residues in the interaction interface. Analyses of a mutant defective in supercomplex formation, which still contains fully functional individual complexes, show that the lack of supercomplex assembly delays the diffusion of cytochrome *c* between the separated complexes, thus reducing electron transfer efficiency. Consequently, competitive cellular fitness is severely reduced in the absence of supercomplex formation and can be restored by overexpression of cytochrome *c*. In sum, our results establish how respiratory supercomplexes increase the efficiency of cellular energy conversion, thereby providing an evolutionary advantage for aerobic organisms.

## Graphical abstract

Mitochondrial respiratory supercomplexes are found in various organisms, however their physiological function remains elusive. This study reveals that supercomplexes enhance mitochondrial electron transfer, resulting in increased competitive fitness.

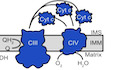

## Introduction

The mitochondrial respiratory chain represents a sophisticated system of multi‐subunit complexes that mediate cellular energy conversion. In mammalian cells, the mitochondrial respiratory chain (MRC) is formed by four distinct complexes (CI–CIV) (Lobo‐Jarne & Ugalde, 2018). Together with the ATP synthase (CV), these complexes form the oxidative phosphorylation (OXPHOS) system, in which electron transfer to molecular oxygen is coupled with the formation of an electrochemical gradient over the inner mitochondrial membrane to drive ATP synthesis (Mitchell, 1966). Given its importance for cellular energy conversion, dysfunction of the OXPHOS system causes various human diseases including neuromuscular and neurodegenerative disorders (Kauppila *et al*, 2017; Kawamata & Manfredi, 2017).

Three major models have been proposed for the structural organization of the MRC: the solid‐state, the liquid‐state (fluid), and the plasticity model (Acín‐Pérez *et al*, 2008; Milenkovic *et al*, 2017; Lobo‐Jarne & Ugalde, 2018). The latter consolidates the previous two models and is based on findings from the past two decades, showing that MRC complexes are not randomly distributed within the inner mitochondrial membrane, but can be assembled into supramolecular structures termed supercomplexes (SCs) (Schägger & Pfeiffer, 2000). Hence, the plasticity model describes a dynamic equilibrium between free complexes and SCs to adapt energy conversion to cellular needs (Acin‐Perez & Enriquez, 2014). SCs exist in a wide range of species, including mammals, plants, yeast, and some bacteria (Milenkovic *et al*, 2017). But despite extensive research, the physiological significance of respiratory SCs remains controversial. Several hypotheses on their role have been suggested. An attractive but highly debated possibility involves SC‐mediated substrate channeling operating such that each SC sequesters and retains its own subpopulation of the mobile electron carriers cytochrome *c* (Cyt *c*) and, for mammalian SCs, ubiquinone (Blanchi *et al*, 2004; Lapuente‐Brun *et al*, 2013). However, no confining structure within SCs is reported to retain Cyt *c* or ubiquinone and accumulating biophysical evidence rather suggests free diffusion of these electron carriers (Blaza *et al*, 2014; Milenkovic *et al*, 2017; Fedor & Hirst, 2018; Hirst, 2018). Further, SCs were suggested to participate in the regulation of respiratory activity (Greggio *et al*, 2017), reduction of oxidative stress (Maranzana *et al*, 2013), as well as assembly and stabilization of individual MRC complexes (Schägger *et al*, 2004; Moreno‐Lastres *et al*, 2012). The latter is based on the observation that defects in CIII or CIV can cause CI deficiency, suggesting a structural interdependence between the affected complexes, which might be mediated by SCs (Acín‐Pérez *et al*, 2004; Schägger *et al*, 2004; Diaz *et al*, 2006). Although it was subsequently reported that cells lacking CIV were shown to still retain significant amounts of assembled CI (Balsa *et al*, 2012), it has been recently disclosed that multiple pathways exist for SC assembly and that the presence of COX1, but not necessarily holo‐CIV, is sufficient to stabilize CI (Lobo‐Jarne *et al*, 2020; Timón‐Gómez *et al*, 2020). CI *ab initio* assembly was described to be only finalized when its nascent form is incorporated into SCs (Moreno‐Lastres *et al*, 2012). However, by using complexome analysis of assembly intermediates in the steady‐state, others suggested that SCs are not required as scaffolding unit, as CI assembly is completed prior to SC formation (Guerrero‐Castillo *et al*, 2017). In addition, SC formation is proposed to prevent protein aggregation in the highly protein‐rich mitochondrial inner membrane (Blaza *et al*, 2014). Several studies have investigated the physiological effects of absence of the mammalian supercomplex assembly factor COX7A2L or SCAF1. Absence of this factor specifically impairs formation of the supercomplex CIII2/CIV, but, importantly, does not impact the respirasomes CI/CIII2/CIVn. In human cells, ablation of COX7A2L has no effect on bioenergetics (Perez‐Perez *et al*, 2016; Lobo‐Jarne *et al*, 2018), but provokes changes in nutrient sensing (Balsa *et al*, 2019). The controversy extents to mouse models, in which energy metabolism effects have been observed or not (Shiba *et al*, 2017; Mourier *et al*, 2014; Lapuente‐Brun *et al*, 2013), and a recent zebrafish model in which abnormal fat deposition was observed (García‐Poyatos *et al*, 2020). Because respirasomes are maintained in these model systems, they do not allow to analyse the physiological relevance to assemble the respiratory chain into higher ordered structures. Hence, several contested hypotheses on the role of SC formation exist, none of which has been sufficiently experimentally evidenced due to the lack of a model system entirely lacking SCs. Thus, the fundamental question remains: What is the purpose of supercomplexes and why do they exist?^1^

## Results and Discussion

### A detailed structure of the CIII–CIV interface allows to design supercomplex‐disrupting mutations

**Figure EV1:**
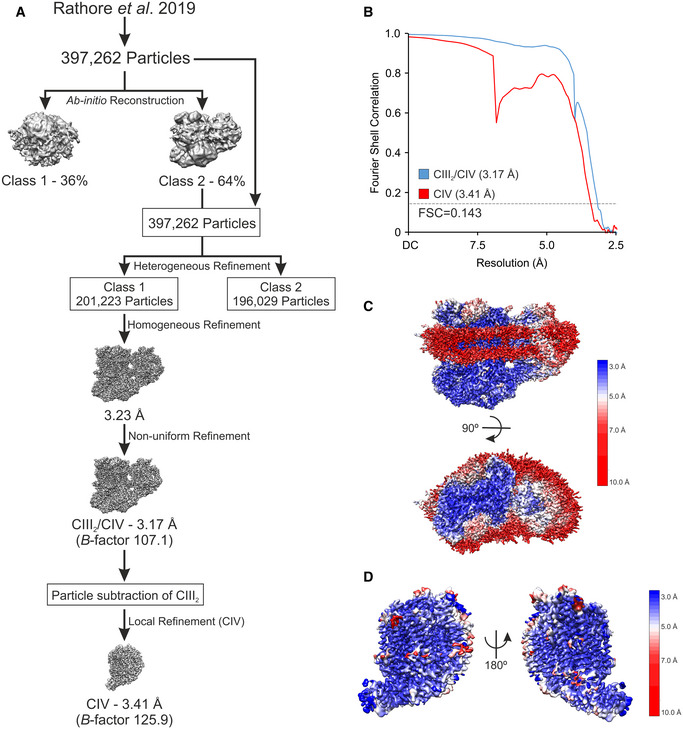
Workflow of cryo‐EM processing ASchematic illustration of Workflow of cryo‐EM processing.BGold‐standard Fourier shell correlation (FSC) of the CIII_2_/CIV supercomplex and the CIV density maps after final refinements. FSC = 0.143 is presented as dashed line.C, DLocal resolution maps of the CIII_2_/CIV supercomplex viewed along the mitochondrial inner membrane (IM) and from the mitochondrial intermembrane space (IMS) side (C), and the CIV density after particle subtraction of CIII_2_ and the digitonin micelle viewed from two rotations along the mitochondrial inner membrane (D).

**Figure 1:**
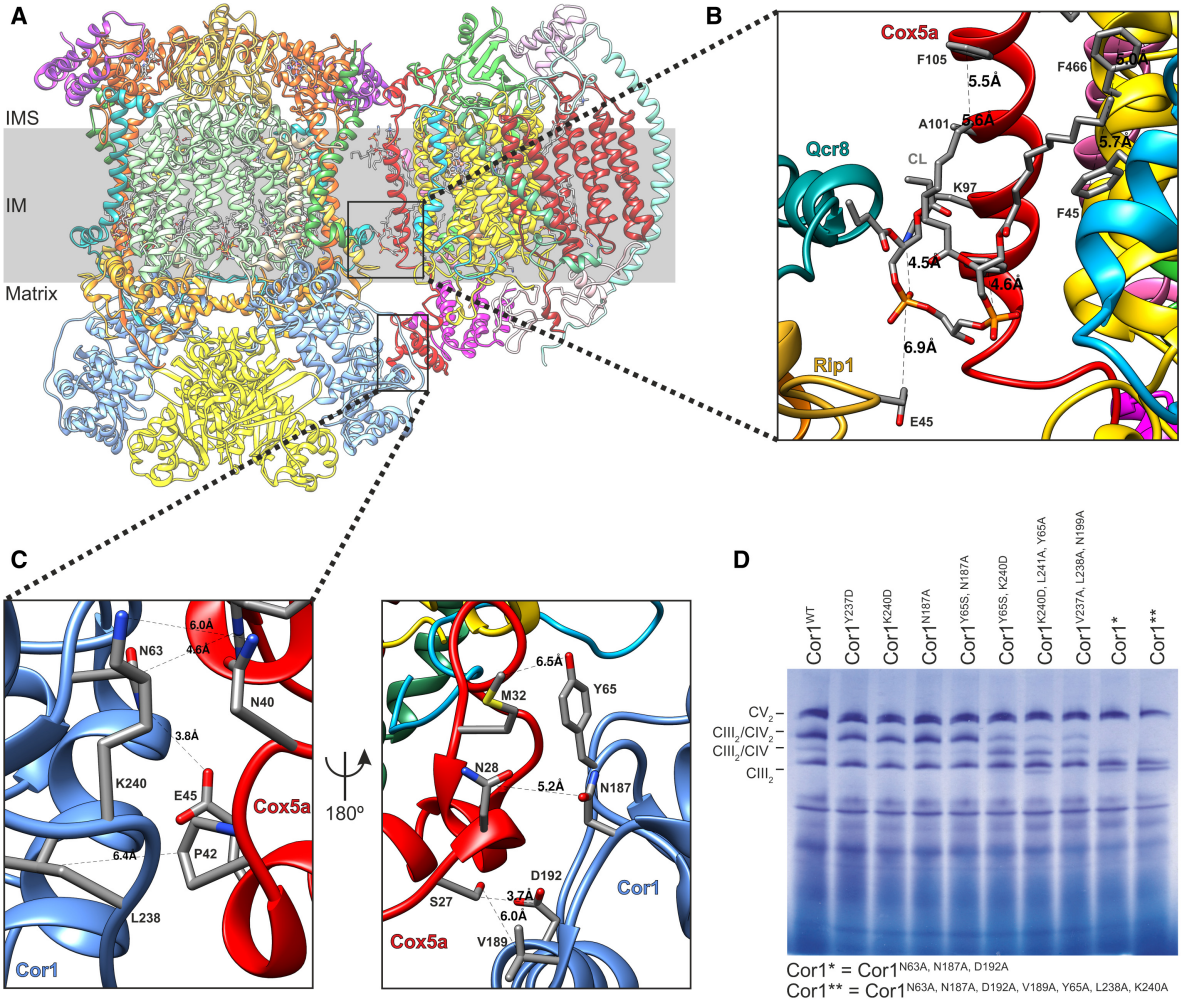
A detailed structure of the CIII‐CIV interface allows to design supercomplex‐disrupting mutations ASide view of the overall structure of the *Saccharomyces cerevisiae* CIII_2_/CIV supercomplex and its position in the mitochondrial inner membrane (IM). IMS: intermembrane space.B, CZoom‐in of the CIII_2_/CIV interaction sites with residues and distances annotated. The inner membrane protein–lipid–protein interactions (Rip1‐CL-Cox5a), where Rip1 is marked in gold, cardiolipin (CL) in gray, and Cox5a in red (B), and the mitochondrial matrix, protein–protein interaction of Cor1 (blue), and Cox5a (red) (C) are shown.DBlue‐native gel electrophoresis of *S. cerevisiae* strains with indicated mutations in Cor1.

**Figure EV2:**
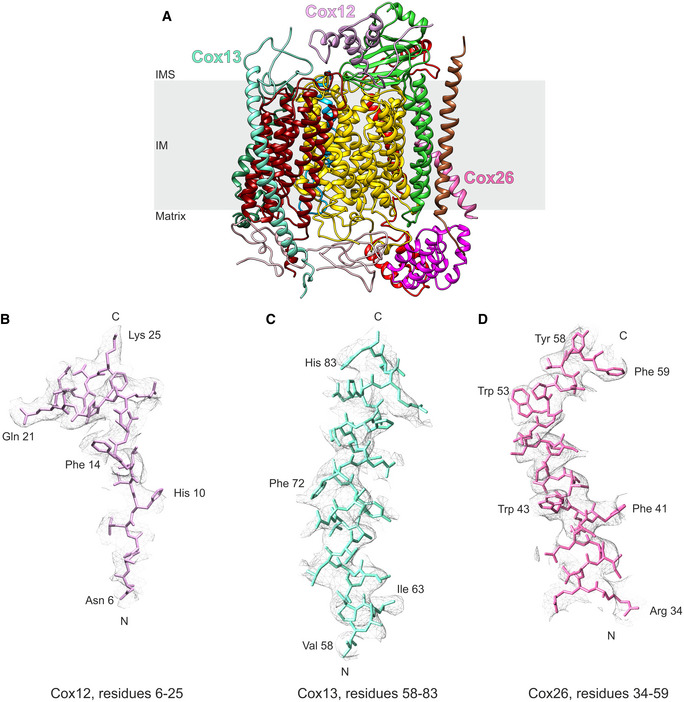
Example densities of CIV subunits Cox12, Cox13, and Cox26 ARibbon diagram of CIV with Cox12, Cox13, and Cox26 annotated. IM: inner membrane; IMS: intermembrane space.B–DExample densities of Cox12 (B), Cox13 (C), and Cox26 (D) demonstrating parts of the subunits that improved due to the re‐processing of the data set.

To address this enigma, we aimed to disrupt SC formation by mutation of key residues in the interaction interface of CIII_2_/CIV SCs of *Saccharomyces cerevisiae*. To this end, we improved the resolution of our previous SC structure (Rathore *et al*, 2019). This yielded a CIII_2_/CIV density map with an overall resolution of 3.17 Å, and, more importantly, after particle subtraction of CIII_2_, the CIV density was solved to an overall resolution of 3.41 Å (Fig EV1A–D, Table EV1). These improvements allowed us to create a more detailed model of several CIV subunits (Figs 1A and EV2A–D). Moreover, we could confirm that the protein–lipid–protein interactions between Cox5a and Rip1 contained cardiolipin (CL) (Fig 1B), as previously suggested (Hartley *et al*, 2019). The improved resolution of the matrix‐localized protein–protein interactions between the CIII‐subunit Cor1 and the CIV protein Cox5a also enabled unequivocal identification of the residues connecting the two complexes (Fig 1C). To gain insights into the physiological function of SCs and explore the consequences of their absence, we performed a variety of alanine‐substitutions in the CIII‐subunit Cor1 to disrupt the CIII–CIV interaction (Fig 1D). Two of these mutants, namely Cor1^N63A, N187A, D192A^ (hereafter Cor1*), and Cor1^N63A, N187A, D192A, Y65A, V189A, L238A, K240A^ (Cor1**), lacked higher molecular weight complexes containing both CIII and CIV, thus revealing complete SC disruption (Fig 1D).

### Characterization of strains lacking respiratory supercomplexes

**Figure 2:**
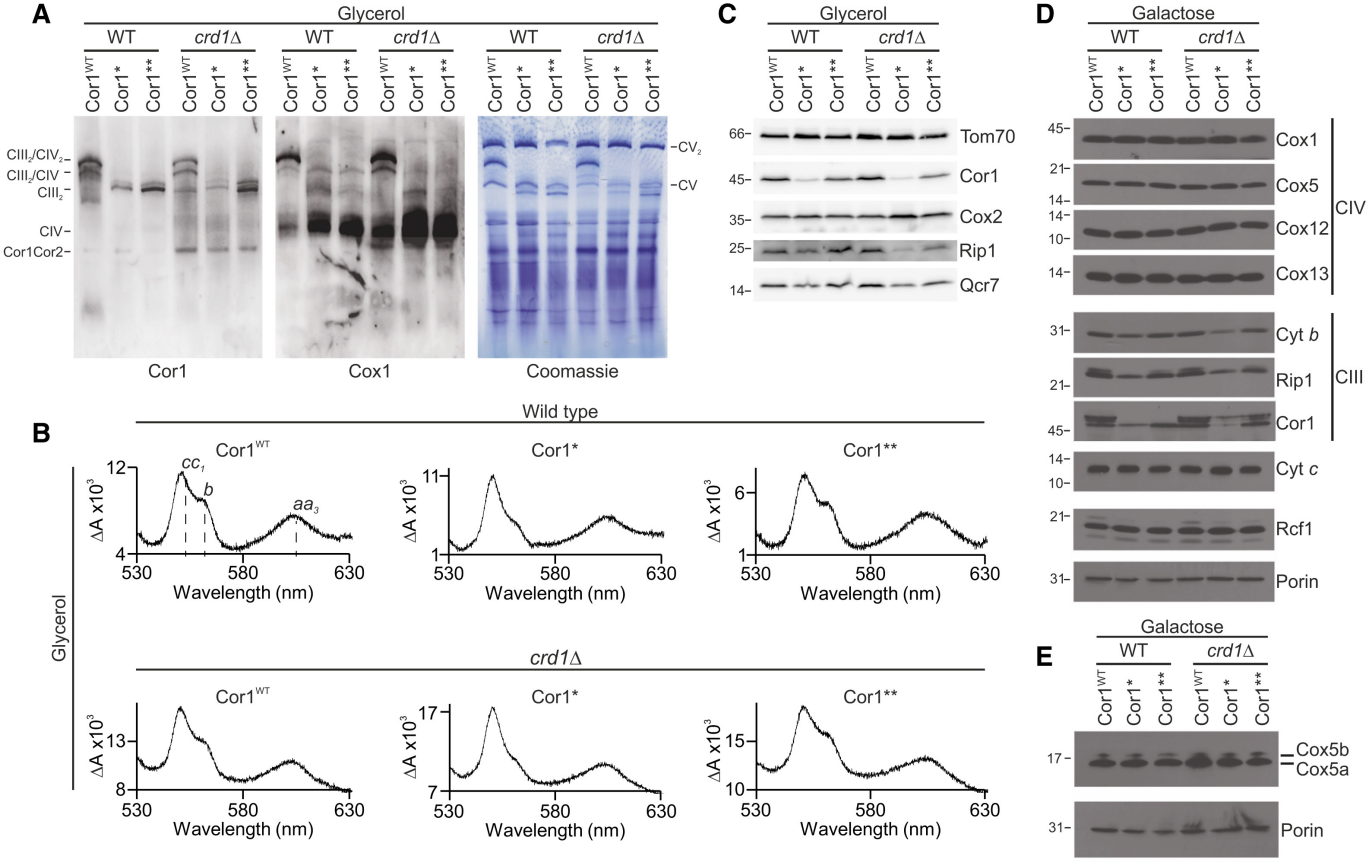
Characterization of strains lacking respiratory supercomplexes ABlue‐native gel electrophoresis and immunoblots of cells expressing the wild‐type form of Cor1 (Cor1^WT^), as well as the mutants Cor1^N63A, N187A, D192A^ (Cor1*) and Cor1^N63A, N187A, D192A, V189A, Y65A, L238A, K240A^ (Cor1**) in wild‐type (WT) background as well as in cells lacking *CRD1* (*crd1*Δ). Blots were probed with antibodies against Cor1 to visualize Complex III (CIII), as well as Cox1, to monitor Complex IV (CIV). CV: Complex V; Cor1Cor2: multimer consisting of Cor1 and Cor2.BReduced‐minus‐oxidized difference spectra of strains described in (A). Heme peaks are indicated for Cor1^WT^ in WT background.C–ESteady‐state protein levels of strains as described in (A) cultivated on YP media containing glycerol (C) or CM media with galactose as carbon source (D, E).

In addition to the Cor1‐Cox5a protein–protein interaction, CIII and CIV also interact with each other through a CL molecule situated between Cox5a and Rip1 (Fig 1B). As CL is thought to be crucial for the structural and functional integrity of SCs (Zhang *et al*, 2002), we generated strains devoid of *CRD1*, encoding cardiolipin synthase. Immunoblotting after BN‐PAGE confirmed that the Cor1 variants lacked SCs also in *CRD1* deletion strains (Fig 2A). However, the absence of CL *per se* did not abolish SC formation in our experimental setup, revealing that the Cor1–Cox5a interaction is necessary and sufficient for forming SCs. Interestingly, CIII_2_ levels were reduced in strains expressing Cor1*, but not in Cor1** (Fig 2A). Likewise, optical spectra analyses revealed a major decrease of heme *b* in the Cor1* strain compared with Cor1 wild‐type (Cor1^WT^) and Cor1** (Fig 2B, Table EV2). Western blot analyses (Fig 2C–E) confirmed reduced levels of Cyt *b* and other CIII subunits (Rip1, Qcr7, and Cor1) in the Cor1* mutant, whereas CIV subunits (Cox1, Cox2, Cox5, Cox12, and Cox13) were not affected (Fig 2C and D). Of note, the Cor1** mutant, despite also being unable to form SCs, had a normal accumulation of CIII subunits. Hence, we decided to focus on the Cor1** strain for further analyses. As Cor1** was sufficient to disrupt SCs and absence of CL did not inhibit SC formation, we also excluded *crd1*Δ strains from further experiments to avoid potential secondary effects caused by the lack of this important mitochondrial phospholipid.

### Abrogation of supercomplexes decreases competitive fitness

**Figure 3:**
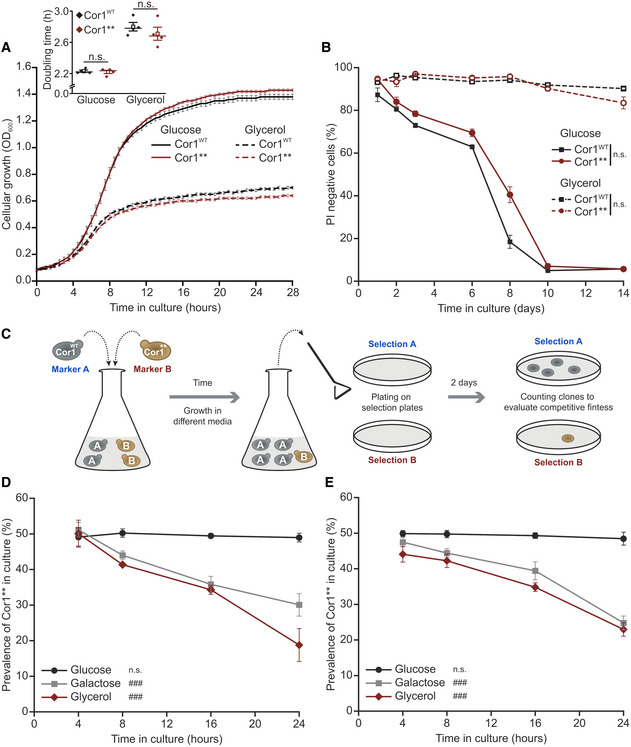
Abrogation of supercomplexes decreases competitive fitness AGrowth analysis of strains expressing the wild‐type form of Cor1 (Cor1^WT^) or the mutant Cor1^N63A, N187A, D192A, V189A, Y65A, L238A, K240A^ (Cor1**). Growth curves and calculated doubling times from the exponential phase (insert) are presented. Cells were cultivated in media containing either glucose or glycerol as carbon source.BDetermination of chronological lifespan via propidium iodide (PI) staining of cells described above.CScheme of the experimental setup to evaluate competitive fitness.D, EAnalysis of competitive fitness as described in (C). Cor1^WT^ strains harbored a chromosomally integrated hygromycin selection cassette, whereas Cor1** contained a clonNAT selection cassette (D). Subsequently, selection markers were switched as a control (E).Data information: For (A, B, D, and E), line graphs indicate mean ± standard error of the mean (s.e.m.). Two‐way ANOVA mixed design followed by Bonferroni *post hoc* test was used to analyze data from 4 individual yeast clones (*n *=* *4 biological replicates). Significances of main effects are visualized as: n.s.: not significant (*P* ≥ 0.05) and ^###^ *P* < 0.001. For insert in (A), data are presented as mean (square) ± s.e.m., median (center line), and single data points (*n *=* *4 biological replicates). Thereby, a two‐tailed independent sample *t*‐test was performed and significances are given as: n.s.: not significant. A detailed description of statistical analyses performed is given in Table EV6.

**Figure EV3:**
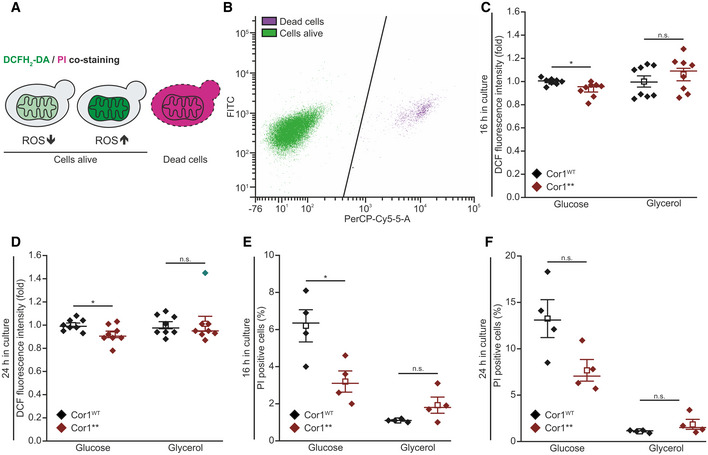
Disruption of supercomplexes does not modulate oxidative stress or cell death A, BScheme of 2′,7′‐dichlorodihydrofluorescein diacetate (DCFH_2_‐DA), and propidium iodide (PI) staining and gating strategy for flow cytometry. PI is taken up by cells upon loss of membrane integrity, thus indicating dead cells. Non‐fluorescent DCFH_2_‐DA is oxidized to fluorescent 2′,7′‐dichlorofluorescein (DCF) upon oxidative stress (A). Hence, the DCF mean fluorescence intensity of PI‐negative cells was applied as a measure for oxidative stress. The threshold used to separate PI‐negative and PI‐positive cells is shown as black line in (B).C, DFlow cytometric quantification of oxidative stress indicated via DCFH_2_‐DA/PI counterstaining of cells expressing the wild‐type form of Cor1 (Cor1^WT^), as well as the mutant Cor1^N63A, N187A, D192A, V189A, Y65A, L238A, K240A^ (Cor1**). Cells were analyzed 16 h (C) and 24 h (D) after inoculation in media either containing glucose or galactose as indicated. To visualize fold values, normalization was performed to Cor1^WT^ cells of the respective medium used.E, FAnalysis of cell death via flow cytometric quantification of PI stained cells as described above. Experiments were performed 16 h (E) and 24 h (F) after inoculation, and the percentages of PI‐positive cells are presented.Data information: Mean (square) ± s.e.m., median (center line), and single data points are depicted (for C and D: *n *=* *8 biological replicates; for E and F: *n *=* *4 biological replicates). Outliers were detected using the 2.2‐fold interquartile range labeling rule and are indicated in turquoise. Two‐tailed independent sample *t*‐tests were used for statistical analysis, except for (C glycerol) and (D glycerol), where two‐tailed Mann–Whitney *U* tests were applied. Significances are visualized as: n.s.: not significant (*P* ≥ 0.05), *P < 0.05. A detailed description of statistical analyses performed is given in Table EV6.

Our engineered Cor1** mutant disrupts SCs formation without affecting individual complexes, thus presenting an advanced tool to study the physiological function of SCs. Hence, we next evaluated the growth rates and chronological lifespan under fermentable (glucose) or respiratory carbon sources (glycerol) in clonal cultures. Unexpectedly, we did not find any significant differences between the Cor1^WT^ and mutant (Cor1**) strains (Fig 3A and B). In line, absence of SC formation had only modest effects on oxidative stress and cell death on glucose and no alterations were observed under respiratory conditions (Fig EV3A–F). Since SC evolved in many different organisms across life kingdoms, we hypothesized that SC formation confers an apparent, selectable advantage. To test this, we determined competitive fitness to mimic a more natural scenario. Hygromycin and clonNAT selection cassettes were chromosomally integrated into Cor1^WT^ and Cor1** cells, respectively, and both strains were inoculated with identical optical density in the same culture. Colony‐forming units on antibiotic selection plates were determined to evaluate the prevalence of either strain in the culture over time (Fig 3C). While no disadvantage of SC disruption was detected during fermentation, growth competition on respiration massively reduced the prevalence of Cor1** in culture (Fig 3D). To avoid potential artefacts, we exchanged the selection markers in a second set of strains and obtained similar results (Fig 3E). We therefore conclude that SC formation results in increased competitive fitness, providing a selectable trait that favored the establishment of SCs during evolution.

### Lack of supercomplexes impairs electron transport between CIII and CIV

**Figure EV4:**
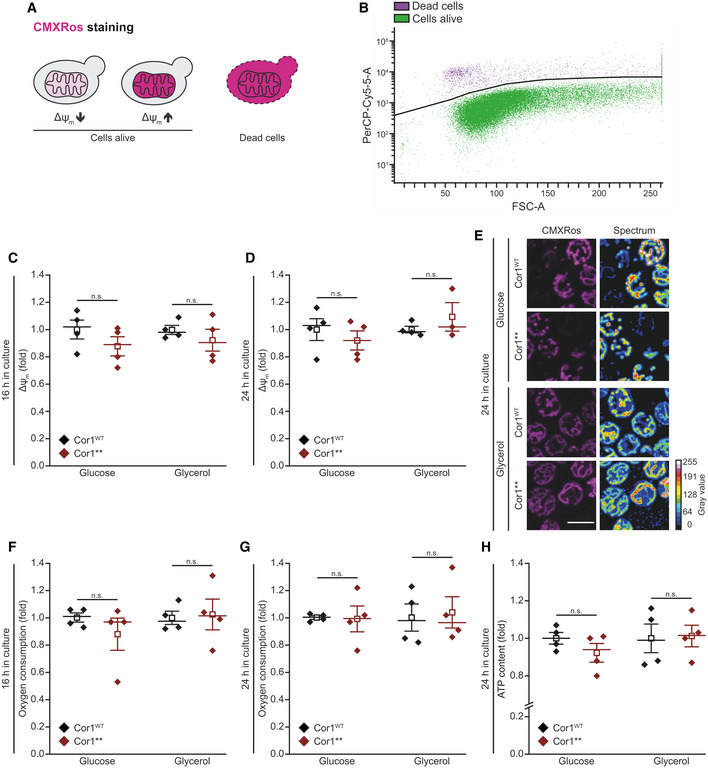
Absence of supercomplex formation does not impact mitochondrial function in living cells A, BScheme of Mitotracker CMXRos staining and gating strategy for flow cytometric analysis. The fluorescence probe is taken up by mitochondria depending on the transmembrane potential (Δψ_m,_). Dead cells accumulate the dye due to a lack of membrane integrity (A) and are excluded from the analysis. The threshold used to separate dead and living cells is shown as black line in (B). The mean fluorescence intensity of cells alive is quantified as a readout for Δψ_m_.C–EAnalysis of Δψ_m_ via Mitotracker CMXRos staining of cells expressing the wild‐type form of Cor1 (Cor1^WT^), as well as the mutant Cor1^N63A, N187A, D192A, V189A, Y65A, L238A, K240A^ (Cor1**). Cells were cultivated in CM media containing indicated carbon sources. Flow cytometric quantification 16 h (C) and 24 h (D) after inoculation are shown, as well as representative confocal micrographs (Z‐projections) after 24 h (E). Normalization in (C) and (D) was performed to Cor1^WT^ cells of the respective medium used.F, GOxygen consumption quantified in intact cells. Strains described in (C) were analyzed 16 h (F) and 24 h (G) after inoculation. Normalization of data was performed as stated in (C) to present fold values.HCellular ATP content measured from cells described in (C). The measurement was performed with cells cultivated for 24 h in CM media containing the indicated carbon sources. Normalization was performed as stated in (C).Data information: Mean (square) ± s.e.m., median (center line), and single data points (*n *=* *4 biological replicates) are depicted. Two‐tailed independent sample *t*‐tests were used for statistical analysis. For (C glycerol), Welch correction was performed. Significances are presented as: n.s.: not significant (*P* ≥ 0.05). A detailed description of statistical analyses performed is given in Table EV6. Scale bar in (E) represents 5 μm.

**Figure 4:**
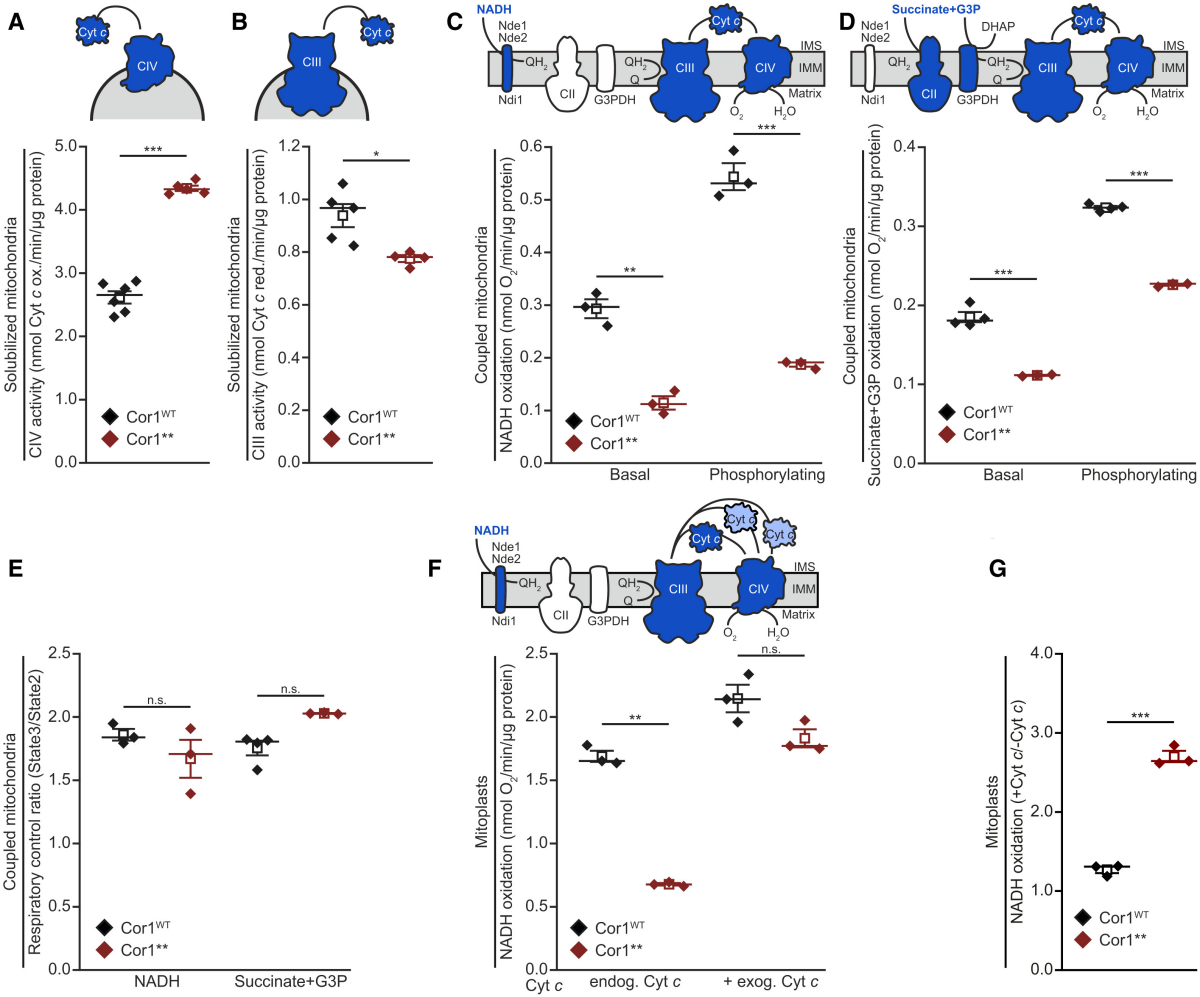
Lack of supercomplexes impairs electron transport from CIII to CIV A, BSpectrophotometric measurement of Cyt *c* oxidase (CIV; A) and NADH Cyt *c* reductase (CIII; B) activities in solubilized mitochondrial extracts. Mitochondria were isolated from cells expressing the wild‐type form of Cor1 (Cor1^WT^) or the mutant Cor1^N63A, N187A, D192A, V189A, Y65A, L238A, K240A^ (Cor1**).C, DPolarographic measurement of KCN‐sensitive oxygen consumption, driven by NADH (C) or succinate + glycerol‐3-phosphate (G3P; D) in isolated coupled mitochondria of indicated strains. Measurements were performed in the absence (basal respiration) or presence (phosphorylating condition) of ADP.ERespiratory control ratio calculated from polarographic measurements of KCN‐sensitive oxygen consumption driven by NADH and succinate + glycerol‐3-phosphate (G3P) in isolated coupled mitochondria of indicated strains.F, GPolarographic measurement of KCN‐sensitive oxygen consumption driven by NADH in mitoplasts in the absence (endog. Cyt *c*) or presence (+ exog. Cyt *c*) of exogenous oxidized Cyt *c*. NADH oxidation (E) and the calculated ratio +Cyt *c*/−Cyt *c* of substrate oxidation (F) are visualized.Data information: Mean (square) ± s.e.m., median (center line) and single data points are depicted. Two‐tailed independent sample *t*‐tests were used to analyze data from at least three individual mitochondrial isolations (*n* ≥ 3 biological replicates), For (A, B, and F endog. Cyt *c*), Welch correction was performed, and for (E Succinate + G3P), a two‐tailed Mann–Whitney *U* test was applied. Significances are given as: n.s.: not significant (*P* ≥ 0.05), *P < 0.05, **P < 0.01, and ***P < 0.001. A detailed description of statistical analyses performed is given in Table EV6.

**Figure EV5:**
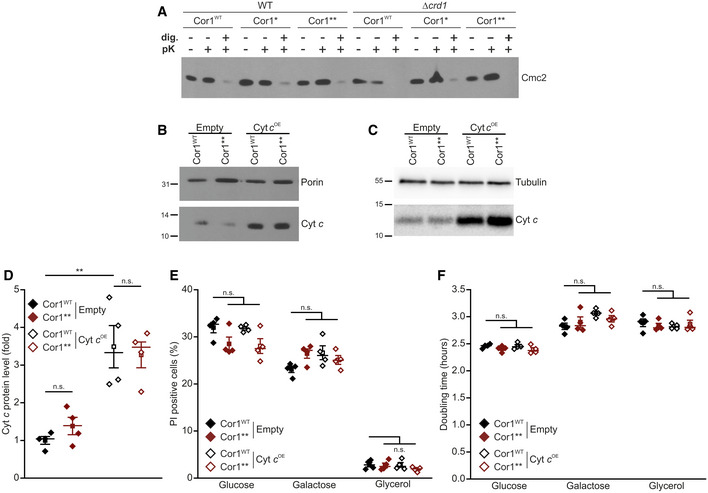
The lack of supercomplexes impairs electron transport from CIII to CIV Assays to verify the intactness of the outer mitochondrial membrane. Mitochondria were isolated from strains expressing the wild‐type form of Cor1 (Cor1^WT^), as well as the mutants Cor1^N63A, N187A, D192A^ (Cor1*) and Cor1^N63A, N187A, D192A, V189A, Y65A, L238A, K240A^ (Cor1**) in wild‐type (WT) background as well as in cells lacking *CRD1* (*crd1*Δ). Intactness of the outer mitochondrial membrane was tested by following the stability of the intermembrane protein Cmc2 1 h after treatment with 6 μg/ml proteinase K (pK). Negative control samples were created by membrane permeabilization with 0.5% digitonin (dig.).Immunoblot analysis of isolated mitochondria from strains expressing the wild‐type form of Cor1 (Cor1^WT^) or the mutant Cor1^N63A, N187A, D192A, V189A, Y65A, L238A, K240A^ (Cor1**). Cells either overexpressed cytochrome *c* (Cyt *c* ^OE^) or contained the empty plasmid (Empty) as a control. Blots were probed with antibodies against Cyt *c*, as well as porin as loading control.Immunoblot analysis as described in (B) from intact cells. Blots were probed with antibodies against Cyt *c*, as well as tubulin as loading control.Densitometric quantification of immunoblots validating overexpression of cytochrome *c* (Cyt *c* ^OE^) in strains expressing either the wild‐type form of Cor1 (Cor1^WT^) or the mutant Cor1^N63A, N187A, D192A, V189A, Y65A, L238A, K240A^ (Cor1**). The Cyt *c* signal was normalized to tubulin intensities as a loading control, followed by normalization to Cor1^WT^ strains harboring the empty vector to present fold values.Flow cytometric quantification of loss of membrane integrity as visualized via propidium iodide (PI) staining of cells described in (D). Cells were cultivated in CM media either containing glucose, galactose or glycerol as carbon source.Doubling time of strains described in (E).Data information: Mean (square) ± s.e.m., median (center line), and single data points (*n *=* *4 biological replicates) are depicted one‐way ANOVA followed by Bonferroni *post hoc* test was applied for statistical analysis, and significances are presented as: n.s.: not significant (*P* ≥ 0.05); **P < 0.01. A detailed description of statistical analyses performed is given in Table EV6.

To characterize the molecular mechanism by which SC formation mediates increased competitive fitness, we evaluated the impact of SC disruption on mitochondrial function. While living cells lacking SCs maintained normal mitochondrial transmembrane potential, oxygen consumption, and ATP levels (Fig EV4A–H), we observed an increased maximal activity of CIV and attenuated CIII activity for the Cor1** mutant in solubilized mitochondria (Fig 4A and B). These results prompted us to investigate electron transfer efficiency of isolated mitochondria (Fig EV5A) utilizing different substrates. When using NADH, which transfers electrons to coenzyme Q via Nde1 and Nde2, but bypasses CII, decreased rates of respiration were detected in both basal (state 2) and ADP‐driven (state 3) conditions (Fig 4C). Similar observations were obtained with a combination of succinate and glycerol‐3‐phosphate (G3P) as substrate, which feed electrons via CII and G3P‐dehydrogenase to coenzyme Q for subsequent delivery to CIII (Fig 4D). Of note, the respiratory control ratios (state 3/state 2), a measure for the coupling between substrate oxidation and phosphorylation, were similar between mitochondria from Cor1^WT^ and Cor1** cells (Fig 4E). We hypothesized that this impairment of the respiratory chain might be caused by inefficient electron transfer from CIII to CIV via Cyt *c*. As neither heme *c* nor Cyt *c* content was reduced in Cor1** mutants compared with Cor1^WT^ expressing cells (Fig 2B and D, and Table EV2), this deficiency in electron transfer is potentially due to increased diffusion distance between the separated MRC complexes. We tested this by analyzing substrate oxidation in mitoplasts (mitochondria with permeabilized outer membrane, thereby exposing the Cyt *c* binding sites to the solvent) with and without administration of exogenous oxidized Cyt *c*. As seen for intact mitochondria, NADH oxidation was severely decreased in mitoplasts derived from Cor1** strains (Fig 4F). Importantly, the addition of exogenous Cyt *c* completely corrected the defect (Fig 4F and G). Taken together, these results demonstrate a decreased efficiency by which Cyt *c* transfers electrons from CIII to CIV upon disruption of respiratory SCs.

**Figure 5:**
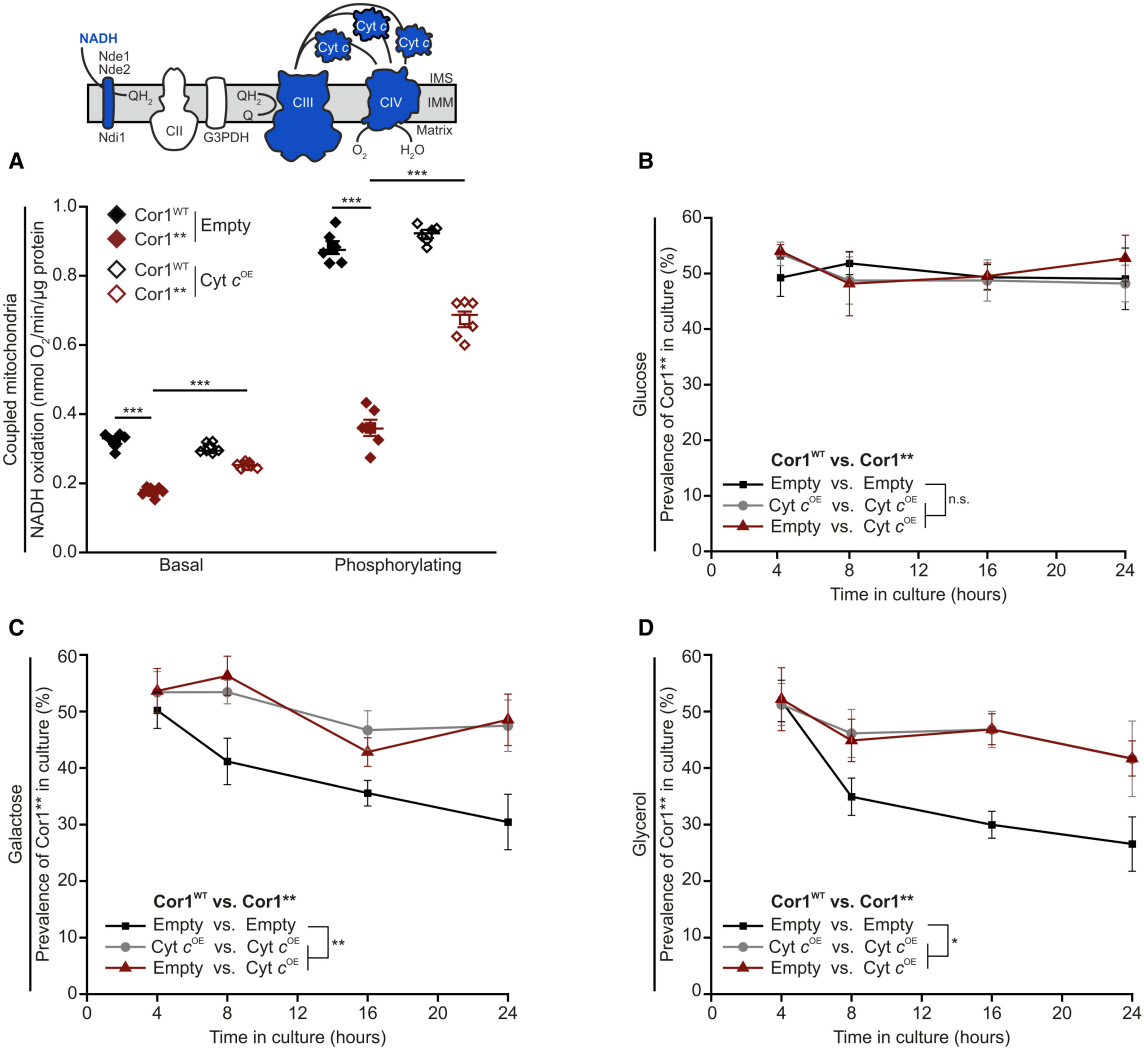
Cytochrome *c* overexpression restores competitive fitness of strains lacking supercomplexes APolarographic measurement of KCN‐sensitive oxygen consumption, driven by NADH in isolated coupled mitochondria. Mitochondria were isolated from cells expressing the wild‐type form of Cor1 (Cor1^WT^) or the mutant Cor1^N63A, N187A, D192A, V189A, Y65A, L238A, K240A^ (Cor1**). Thereby, strains either overexpressed cytochrome *c* (Cyt *c* ^OE^) or harbored the empty plasmid as a control (Empty). Measurements were performed in the absence (basal respiration) or presence (phosphorylating condition) of ADP.B–DAnalysis of competitive fitness of strains described in (A). Cor1^WT^ strains harbored a chromosomally integrated clonNAT selection cassette, whereas Cor1** contained a hygromycin selection cassette. Strains were cultivated in CM media containing glucose (B), galactose (C), or glycerol (D) as carbon source.Data information: For (A), mean (square) ± s.e.m., median (center line), and single data points (*n *=* *6 biological replicates) are depicted, and an one‐way ANOVA followed by a Bonferroni *post hoc* test was used for statistical analysis. For (B–D), mean ± s.e.m. (*n *=* *4 biological replicates) are depicted, and data were statistically evaluated by two‐way ANOVA mixed design followed by a Bonferroni *post hoc* test. Significances are visualized as: n.s.: not significant (*P* ≥ 0.05), *P < 0.05, **P < 0.01, ***P < 0.001. A detailed description of statistical analyses performed is given in Table EV6.

Next, we tested whether an increase in endogenous Cyt *c* levels can restore electron transfer in intact mitochondria in a similar way as observed upon exogenous administration of Cyt *c* to mitoplasts. We therefore isolated mitochondria from cells overexpressing Cyt *c* (Fig EV5B). Indeed, higher levels of Cyt *c* could restore NADH oxidation rates in strains lacking SCs at both basal and phosphorylating conditions (Fig 5A). This therefore showed that increased levels of Cyt *c* can compensate for the decreased electron transfer efficiency upon SC disruption. Inspired by these results, we next set out to test whether increased level of Cyt *c* can also restore competitive fitness of cells lacking SCs. In intact cells, overexpression of Cyt *c* led to a 3.5‐fold increase in protein level compared to control cells (Fig EV5C and D). Despite being a pro‐apoptotic factor upon release into the cytosol in both yeast and mammals (Guaragnella *et al*, 2012), Cyt *c* overexpression did not significantly affect cellular growth rates nor cell death within 24 h after inoculation (Fig EV5E and F). This allowed us to determine whether increased Cyt *c* protein levels can correct the reduced competitive fitness upon SC disruption. Indeed, elevated Cyt *c* levels restored competitive fitness defects in the Cor1** cells on non‐fermentable carbon sources (Fig 5B–D). In aggregate, our data demonstrate that SC formation mediating close proximity between individual complexes allows efficient electron transfer via Cyt *c*. Thereby, SC formation constitutes a fitness advantage that enforced the formation of these macromolecular structures during evolution.

Since the discovery of mitochondrial respiratory SCs two decades ago, several hypotheses on their functional and physiological relevance have been suggested, including increased electron transfer rates through substrate channeling (Blanchi *et al*, 2004; Lapuente‐Brun *et al*, 2013). However, biophysical evidence argues against strict substrate channeling (Blaza *et al*, 2014; Fedor & Hirst, 2018). Accordingly, the decreased coupling efficiency identified in our work cannot be caused by an impairment of a strictly defined substrate channeling operating by Cyt *c* trapped in a SC. The distance between the respective Cyt *c* binding sites between CIII and CIV in SC configuration (70 Å in yeast SC as compared to 100 Å in the mammalian respirasome) renders direct electron transfer via one single bound Cyt *c* molecule impossible (Rathore *et al*, 2019). Importantly, our data are in line with a scenario where electron transfer by Cyt *c* between CIII and CIV is more efficient in the SC configuration than between the separated complexes (Trouillard *et al*, 2011; Hirst, 2018; Stuchebrukhov *et al*, 2020). Hence, our data comply with a scenario where the main physiological function of SCs is to bring individual MRC complexes in close proximity, allowing a more efficient electron transfer via the mobile electron carriers between the complexes.

Our data further show that this rather simple function of SCs has extensive implications for the evolution of aerobic organisms. In clonal cultures, the absence of SC formation did not provoke a massive defect in several physiological parameters, including ATP levels, oxidative stress, or chronological life span, thereby disputing the suggestion that SCs play a role in reducing reactive oxygen species (Maranzana *et al*, 2013). Thus, the electron transfer deficiency caused by SC disruption does either not exceed a critical threshold that would result in an detectable OXPHOS impairment, or it might be counteracted by an upregulation of protective genetic programs to compensate cytotoxicity (Melber & Haynes, 2018; Suhm *et al*, 2018). In both cases the formation of SCs presents the favorable solution, explaining why these structures arose during evolution. The main driving force of evolution is the competition for resources. In the case of yeast in its natural habitat, this involves competition with other yeast strains and bacteria that populate the same environment. Cells that can use the actual conditions best and consequently contain the most efficient pathways to exploit certain substrates will prevail. Indeed, when applying competitive fitness analyses cells lacking SCs were outcompeted only under conditions requiring respiration, an effect that could be compensated by overexpression of Cyt *c*. This illustrates that the increased electron transport efficiency, which enhances OXPHOS, is an evolutionary advantage favouring the formation of SCs.

In summary, our results demonstrate that the formation of SCs is important for efficient electron transfer between CIII and CIV by positioning the Cyt *c* binding sites of both complexes in close vicinity, a conserved feature of SC architecture found in multiple organisms. Thereby, SC formation confers a competitive fitness advantage for aerobic organisms, which constituted a selectable trait during evolution.

## Material and Methods

### Yeast strains used in this study

All stains in this study were isogenic to the intronless W303 strain MRSI^0^ (Gruschke *et al*, 2011) and are listed in the Table EV3. *COR1* and *CRD1* were disrupted with homologous recombination using a Kanamycin resistance cassette and a *TRP1* selection cassette, respectively. The primers used for this approach (*COR*1_fw; *COR*1_rev, *CRD*1_fw and *CRD*1_rev) are described in Table EV4. Cor1 mutations were ordered from GeneArt Gene Synthesis (Thermo Fisher) and cloned into a pRS305 integrative plasmid, where the variants of the *COR1* gene were inserted together with 300 bp upstream and 200 bp downstream of the open reading frame using the restrictions sites *Bam*HI and *Sal*I. *COR1‐*containing plasmids were linearized with *Afl*II and integrated into the *LEU2* locus of the genome with lithium acetate transformation method (Gietz & Woods, 2002). For overexpression of yeast Cyt *c* in experiments evaluating competitive fitness, the *CYC1* gene was amplified from yeast genomic DNA (primers: *CYC*1_fw and *CYC*1_rev) and cloned into a pCM190 plasmid using a Gibson Assembly Cloning Kit (New England BioLabs; E5510S) following the supplier's protocol. Thereby, pCM190 was amplified with the primers pCM190_fw and pCM190_rev. For the biochemical experiments, the *CYC1* gene under the control of its endogenous promoter was sub‐cloned in the episomal plasmid YEplac112 as a *Xma*I‐*Hind*III fragment from the Yep351‐*CYC1* (ST13) construct reported in (Barrientos *et al*, 2003).

### Culture conditions

Cells were grown in full media (YP; 1% yeast extract, 2% peptone) or complete minimal (CM) media (0.17% yeast nitrogen base, 0.5% (NH_4_)_2_SO_4_, 30 mg/l of all amino acids—except 80 mg/l histidine and 200 mg/l leucine—30 mg/l adenine and 320 mg/l uracil), as indicated. Media were supplemented with respective carbon sources (2% glucose, 2% galactose or 2% glycerol), and 2% agar was added for solid media. Where applicable, 300 mg/l hygromycin B or 100 mg/l nourseothricin was added after autoclaving for selection.

For assessing cellular consequences of SC disruption, overnight cultures were grown for 16–20 h at 28°C, 145 rpm in CM media containing glucose in glass eprouvettes and used for inoculating the main culture in 10 ml CM medium in baffled 100 ml Erlenmeyer flasks to an OD_600_ of 0.1. Samples were taken at indicated time points and further processed as described below. Of note, at least four different clones per strain were applied for the analysis to consider clonogenic variation. To suppress Cyt *c* overexpression in respective strains, doxycycline (40 μg/ml) was added to agar plates and expression was induced by inoculation of cells in media without doxycycline.

### Cryo‐EM image processing

Particles from a dataset previously acquired (Rathore *et al*, 2019) were re‐processed using an updated version of the software cryoSPARC (Punjani *et al*, 2017) (Fig EV1A; Table EV1). In brief, 397,262 particles with a calibrated pixel size of 1.06 Å per pixel were imported into cryoSPARC v2.2. An initial model of the CIII_2_/CIV respiratory SC was created using 253,478 particles in the *ab initio* function of the software before all the particles were 3D‐classified using the *heterogeneous refinement* tool with a low‐pass filtered (30 Å) version of that model as a reference. This resulted in a class of 201,223 particles that we continued to use. *Non‐uniform refinement* of these particles generated a 3.17 Å density map (GSFSC = 0.143; B‐factor: −107.1; Fig EV1B), which was used to model the CIII_2_/CIV respiratory SC. From the atomic coordinates of CIII_2_, and CIV, two masks with a resolution of 7 Å and 3 pixels extension (6 pixel soft edge) was created using RELION3 (Zivanov *et al*, 2018) and imported to cryoSPARC. Using the *particle subtraction* tool and *local refinement* tool to subtract the signal from CIII_2_ and refine CIV alone, we could generate a 3.41 Å density map (GSFSC = 0.143; B‐factor: −125.9; Fig EV1B), which was used to model the atoms of CIV. Using cryoSPARCs *local resolution* function (FSC = 0.5), we estimated the local resolution for both classes (Fig EV1C and D).

### Model building, validation, and figure preparation

The 3.17 Å (FSC = 0.143) density map of the CIII_2_/CIV SC was used for model building the SC in Coot v0.8.8 (Emsley *et al*, 2010). The previous structure of the CIII_2_/CIV subunit [PDB: 6GIQ; (Rathore *et al*, 2019)] was used as a starting reference for all model building. The amino acid positions of both subunits were manually corrected to the new density in Coot after the reference had been rigid‐body fitted to the new map. Additional improvement of the CIV subunits was built using the 3.41 Å (FSC = 0.143) density map. The phospholipid in the interface previously described could here be identified and placed correctly. Restrained model refinement was done with PHENIX v1.13 *phenix.real_space_refine* (Liebschner *et al*, 2019) with additional restraints applied for all ligands. These restraints were made using the *eLBOW* function in PHENIX. First, the separate CIII_2_ and CIV subunits were refined individually before being combined to perform the final refinement. Settings applied for the rounds of global real‐space refinement were as follows: five macro cycles with secondary‐structure, rotamer, Ramachandran, and Cβ‐torsion restraints. The final model was inspected again in Coot and validated using MolProbity (Chen *et al*, 2010). Additional refinement statistics are provided in Table EV1.

All figures of the cryo‐EM maps and atomic models were made in UCSF Chimera v1.13.1 (Pettersen *et al*, 2004), as well as UCSF ChimeraX (Goddard *et al*, 2018) and processed with CorelDRAW X8.

### Isolation of mitochondria

Mitochondria were isolated according to (Meisinger *et al*, 2006) with slight modifications. In brief, yeast cells were grown either in CM media supplemented with galactose or YP media, containing glycerol to early‐ or mid‐exponential phase (OD_600_ ≈ 0.8 or 2.0), respectively. Cells were harvested by centrifugation (3,000 *g*, 5 min) and washed once with distilled water before being resuspended (2 ml/g cell wet weight) in MP1 buffer (0.1 M Tris, 10 mM dithiothreitol, pH 9.4) and incubated for 10 min at 30°C. Cells were then washed with 1.2 M sorbitol, before being resuspended (6.7 ml/g wet cell weight) in MP2 buffer (20 mM potassium phosphate, 0.6 M sorbitol, pH 7.4), containing 3 mg/g of cell wet weight zymolyase 20T. Spheroplasts were created by incubation for 1 h at 30°C and harvested by centrifugation (3,000 *g*, 5 min, 4°C). After careful resuspension in 13.4 mg/g of cell wet weight in ice‐cold homogenization buffer (10 mM Tris, 0.6 M sorbitol, 1 mM ethylenediaminetetraacetic acid, 1 mM phenylmethylsulfonyl fluoride, pH 7.4), homogenization was performed by ten strokes with a Teflon plunger (Sartorius Stedim Biotech S.A.). Homogenates were centrifuged at 3,000 *g* for 5 min at 4°C, and the supernatants were subsequently centrifuged at 17,000 *g* for 12 min at 4°C. Pelleted mitochondria were resuspended in isotonic buffer (20 mM HEPES, 0.6 M sorbitol, pH 7.4) to a concentration of 10 mg/ml.

The intactness of the outer mitochondrial membrane was tested by following the stability of the intermembrane protein Cmc2 2 h after treatment with 6 μg/ml proteinase K. Negative control samples were created by membrane permeabilization with 0.5% digitonin.

### Blue‐Native PAGE and immunoblot analysis

Isolated mitochondria (100 μg) were centrifuged at 16,000 *g* for 10 min at 4°C, and the resulting pellet was resuspended in 15 μl lysis buffer (50 mM Bis‐Tris, 100 mM KCl, 2 mM Aminohexanoic acid, 1 mM EDTA, 1× Complete Protease Inhibitor cocktail (Roche), 1 mM phenylmethylsulfonyl fluoride (PMSF), 12% glycerol, 2% digitonin, pH 7.2) and incubated for 10 min on ice. After centrifugation (16,000 *g*, 10 min, 4°C), 1.5 μl of sample additive (5% G‐250) was added, and the samples were loaded on a 3–12% precast native gel (Invitrogen). For subsequent immunoblot analysis, a transfer on PVDF membranes was conducted for 2 h at 200 mA.

For the analysis of steady‐state protein levels, cells were harvested when reaching OD_600_ of 1.5, resuspended in 0.1 NaOH and incubated for 5 min at room temperature (RT). After centrifugation (16,000 *g*, 5 min, RT), the pellet was resuspended in 50 μl of SDS sample buffer (63 mM Tris, 2% SDS, 10% glycerol, 0.1% β‐mercaptoethanol and 0.0005% bromophenol blue, pH 6.8) and incubated for 3 min at 95°C. After centrifugation (14,000 *g*, 2 min, RT), 25 μl of the supernatants was applied for standard SDS–PAGE. All antibodies used in this study are listed in the Table EV5.

### UV‐VIS spectroscopy

Optical spectra (400–650 nm) of isolated mitochondria were recorded using a Cary4000 UV‐Vis spectrophotometer (Agilent Technologies). In brief, 200 μg of isolated mitochondria grown in YP media containing glycerol were lysed in 20 μl of lysis buffer (50 mM KPi pH 7.4, 150 mM KCl, 1× Complete Protease Inhibitor cocktail (Roche), 1 mM PMSF, 1% *n*‐Dodecyl β‐D‐maltoside) for 20 min at 4°C, before being mixed with 130 μl of dilution buffer (50 mM KPi pH 7.4, 150 mM KCl) in a microcuvette and measured with a spectrophotometer. To get the reduced spectra, a small amount of sodium dithionite was added to the cuvette before the spectra were measured again. Heme concentrations were determined from the differential spectrum (reduced‐minus‐oxidized) from three separate measurements and preparations. The concentration of *a*‐type hemes (*aa*_3_) was determined by applying the absorption coefficient ε = 23.2/mM/cm (Vanneste, 1966) at ΔA^605^, using a linear trend line between 577–630 nm to calculate the base of the peak. Similarly, the bases of heme *b* (ΔA^562^) and the c‐type hemes *cc*_1_ (ΔA^553^) peaks were determined from a trend line between 540–577 nm. The concentrations of these hemes were determined as previously described (Guergova‐Kuras *et al*, 1999), using the formulas:

$$\left[\text{heme}\;b\right]\left(\text{mM}\right)=\Delta A^{562}\times 3.539\times 10^{-2}-\Delta A^{553}\times 1.713\times 10^{-3}$$

$$\left[\text{heme}\;cc_{1}\right]\left(\text{mM}\right)=\Delta A^{562}\times 5.365\times 10^{-2}-\Delta A^{553}\times 9.564\times 10^{-3}$$

To separate heme *c* from the heme *cc*_1_ peak, mitoplasts were created by swelling the mitochondria in hypotonic buffer (50 mM KPi pH 7.4) for 30 min, before adjusting its concentration of KCl to 500 mM. Mitoplasts were pelleted (25,000 *g*, 10 min, 4°C), and the supernatant was used for spectroscopic measurements as described above. Heme *c* concentrations were determined by applying the absorption coefficient ε = 21.8/mM/cm using a trend line between 540–577 nm to calculate its base peak.

### Polarographic measurement of substrate oxidation

Using a Clark‐type oxygen electrode (Hansatech Instruments, Norfolk, UK), KCN‐sensitive oxidation of different substrates was analyzed in isolated mitochondria as described recently (Ocampo *et al*, 2010). Coupled mitochondria (30 μg) were resuspended in 750 μl isotonic respiration buffer (0.6 M mannitol, 20 mM HEPES pH 7.0, 10 mM H_3_PO_4_ pH 7.4, 2 mM MgCl_2_, 1 mM EGTA, and 0.1% BSA) and transferred into the polarographic chamber, which was subsequently supplemented with respiratory substrates (10 μl of 0.1 M NADH, 10 μl of 0.4 M succinate + 10 μl of 0.5 M glycerol‐3‐phosphate, or 9.4 μl of 0.4 M ascorbate + 6 μl of 12 mM N,N,N′,N′‐tetramethyl‐p‐phenylenediamine (TMPD)). Following the measurement of state 2 respiration, 10 μl of 20 mM ADP was admixed, and state 3 respiration was validated. Three μl of 80 mM KCN solution were finally added to assess the specificity of the assay. To analyze maximum uncoupled respiration, similar experiments were performed in hypotonic respiratory buffer containing 10 mM H_3_PO_4_ (pH 7.4) and 1 mM EDTA. In some experiments performed in these conditions, oxidized cytochrome *c* (3 μl of 1% solution (*w*/*v*)) was added to assess exogenous cytochrome *c*‐stimulated respiration. To control for the complete oxidation of our cytochrome *c* stock, we verified that no increase in respiration occurred when cytochrome *c* was added to uncoupled mitochondria in the absence of substrates.

### Spectrophotometry of respiratory chain activity

Isolated mitochondria were also used for spectrophotometric assays performed at 24°C to measure KCN‐sensitive cytochrome *c* oxidase (CIV) activity and antimycin A‐sensitive cytochrome *c* reductase (CIII) activity, as described in (Barrientos *et al*, 2009). Briefly, CIII enzymatic activity was measured in 1 ml 20 mM phosphate buffer pH 7.4 containing 0.05% oxidized cytochrome *c*, 0.4 μM KCN and 50 μM decyl‐ubiquinol (coenzyme Q reduced with lithium borohydride). The reaction was started by addition of approximately 30 μg of mitochondrial proteins permeabilized with final 0.5% sodium deoxycholate. Addition of the CIII inhibitor antimycin A (80 μM final concentration) was used to test reaction specificity. Similarly, CIV enzymatic activity was measured in 1 ml 20 mM phosphate buffer pH 7.4 containing 0.08% sodium dithionite‐reduced cytochrome *c*. The reaction was started by addition of approximately 20 μg of mitochondrial proteins permeabilized with final 0.5% sodium deoxycholate. Addition of the CIV inhibitor KCN (0.24 μM final concentration) was used to test reaction specificity. For both reaction, absorption was recorded at 550 nm wavelength and specific activities were calculated using the cytochrome *c* extinction coefficient at 550 nm of 18.5/mM/cm.

### Analysis of cellular growth

Cellular growth was analyzed with a Bioscreen CTM automated microbiology growth curve analysis system (Growth Curves, USA). Overnight cultures were grown as described above and used for inoculation in CM media containing indicated carbon sources to an OD_600_ of 0.1 in the suppliers “honeycomb microplates”. Thereby, a final volume of 250 μl per well was used and automatically measured every 30 min at 28°C and shaking at the maximum level. The respective medium without cells was applied as blank, and doubling time was calculated from growth curves during the logarithmic growth phase.

### Analysis of oxidative stress and cell death

Oxidative stress was flow cytometrically quantified by simultaneous staining of cells with 2′,7′‐dichlorodihydrofluorescein diacetate (DCFH_2_‐DA; Sigma) and propidium iodide (PI; Sigma). Approximately 2 × 10^6^ cells were collected by centrifugation in 96‐well plates and resuspended in 250 μl phosphate‐buffered saline (PBS, 25 mM potassium phosphate, 0.9% NaCl, pH 7.2) containing 100 μg/l PI and 100 mM DCFH_2_‐DA. PI is a fluorescence dye that is taken up in cells upon loss of membrane integrity, indicating cell death, and non‐fluorescent DCFH_2_‐DA is oxidized by reactive oxygen species and nitric oxide to fluorescent 2′,7′‐dichlorofluorescein (DCF), indicating overall oxidative stress. Cells were incubated for 10 min at RT in the dark, washed once in PBS, and 30,000 cells per sample were analyzed via a BD LSR Fortessa and FACSDivia software. Mean fluorescence intensity of the DCF from PI‐negative cells was evaluated as a readout for pre‐lethal oxidative stress and was normalized to control cells expressing the wild‐type form of Cor1 (Cor1^WT^) on respective media to present fold values. For quantification of cell death, the percentage of PI‐positive cells in the culture is visualized.

### Analysis of mitochondrial transmembrane potential

Assessment of mitochondrial transmembrane potential (Δψ_m_) of intact cells was performed according to (Aufschnaiter *et al*, 2018) with slight adaptations. In brief, approximately 2 × 10^6^ cells were harvested in 96‐well plates and resuspended in 250 μl PBS containing 5% glucose and 200 nM Mitotracker CMXRos (Thermo Fisher Scientific). Cells were incubated for 10 min at RT in the dark, washed once in PBS, and analyzed by flow cytometry as described above. Dead cells that accumulated the dye due to a loss of membrane integrity were excluded from the analysis, and the mean fluorescence intensity of Mitotracker CMXRos of the remaining population was used as a readout for Δψ_m_. Normalization to Cor1^WT^ was done as described for DCFH_2_‐DA/PI staining.

### Confocal microscopy

For monitoring Δψ_m_ via confocal microscopy, the specimens were prepared on agar slides to immobilize yeast cells after staining as described above. Samples were monitored with a Leica SP5 confocal laser scanning microscope, equipped with a Leica HCX PL Apo 63× NA 1.4 oil immersion objective. To efficiently monitor the 3‐dimensional mitochondrial network, *Z*‐stacks were recorded using 64 × 64 × 12.6 (*x*/*y*/*z*) nm sampling and Z‐projections were created with the open‐source software Fiji (Schindelin *et al*, 2012). Thereby, Gaussian filtering (*x*σ = *y*σ = *z*σ = 1), followed by background subtraction (rolling ball radius = 50 pixels) was conducted, and the pictures were visualized with the maximum‐intensity projection method. The dynamic range of presented figures was adapted by using the “Brightness/contrast” tool of Fiji. All pictures within an experiment were captured and processed with the same settings.

### Measurement of oxygen consumption in intact cells

Oxygen consumption of intact yeast cells was investigated with a Fire‐Sting optical oxygen sensor system (Pyro Science) as described recently (Aufschnaiter *et al*, 2018). Oxygen sensor spots were mounted on 2 ml clear glass vials, filled with culture, hermetically sealed, and immediately used for analysis. Oxygen concentration in the vials was measured for 1 min, and the slope of the regression line was calculated. In parallel, the percentage of cells alive was determined via flow cytometric analysis of PI stained cells (as described above), and the total number of cells per sample was quantified with a CASY cell counting device (Schärfe Systems). These parameters were used to normalize the quantified oxygen consumption to the number of cells alive in the sample, which is expressed as fold change compared with Cor1^WT^ cells cultivated in respective media.

### Determination of cellular ATP content

Cellular ATP levels were quantified with a luminescent ATP detection assay kit (Abcam) as described recently (Aufschnaiter *et al*, 2018). Thereby, 1 ml of culture was harvested, and ATP was extracted with the hot ethanol method, where cells were flash‐frozen in liquid nitrogen, resuspended in 0.5 ml BES buffer (75% EtOH, 10 mM (NH_4_)_2_SO_4_) and incubated for 3 min at 90°C. After centrifugation for 20 min, 4°C with 16,000 *g*, samples were diluted 20‐fold in Tris buffer (20 mM, pH 8), and 150 μl were transferred into a low‐bind 96‐well plate and incubated for 5 min for adaption to RT. 50 μl of the supplied substrate solution were added in each well, and the plate was placed into a GloMax Multi detection system (Promega) for 10 min for incubation and dark adaption. The luminescence signal was then measured via integration over 10 s. Normalization to cells alive via PI staining and cell counting and calculation of fold values was done as described above for oxygen consumption assays.

### Analysis of competitive fitness

For competitive fitness assays, a Hygromycin selection cassette was amplified from a pFA6a‐hphNT1 plasmid (Janke *et al*, 2004) with the primer *HIS3*_fw and *HIS3*_rev (listed in Table EV4) and integrated via homologous recombination into the *HIS3* locus of Cor1^WT^ and Cor1**. Similarly, the Nourseothricin (clonNAT) selection cassette was amplified from pFA6a‐natNT2 (Janke *et al*, 2004) with the same primer set and integrated into the *HIS3* locus of Cor1^WT^ and Cor1**. Separated overnight cultures of each strain (Cor1^WT^ with Hygromycin resistance and Cor1* with clonNAT resistance) were used to inoculate a mixed culture with OD_600_ of 0.1 per strain. Thereby, CM media was used with indicated carbon sources. The mixed cultures were grown at 28°C, 145 rpm, and aliquots were taken at indicated time points and plated on both Hygromycin and clonNAT containing YP agar plates with glucose. After 2 days of incubation, colony‐forming units were counted and the prevalence of Cor1** was calculated as the percentage of colony‐forming units with resistance against clonNAT compared with colony‐forming units on Hygromycin and clonNAT containing agar plates. Of note, this experiment was conducted with a second set of strains, where selection markers were switched (Cor1^WT^ resistant against clonNAT, Cor1** resistant against Hygromycin) to avoid artefacts mediated by the different selection cassettes.

### Statistical analysis

Results are presented as line graphs, indicating mean ± standard error of the mean (s.e.m.), or dot plots with mean (square) ± s.e.m. and median (center line), as well as single data points. The number of *n* represents the amount of individual clones or mitochondrial preparations used for the analysis and is indicated in respective figure legends. Outliers were defined as data points outside the 2.2‐fold interquartile range and are highlighted in turquoise. Upon the presence of outliers, alternative non‐parametric tests were performed (for a detailed description of the statistical analysis of each experiment performed see Table EV6). The normal distribution of the data was assessed with a Shapiro–Wilk's test, and the homogeneity of variances was evaluated with a Levene's test (both analyzed with OriginPro 2020, OriginLab). A detailed description of the procedure upon violation of respective assumptions is presented in Table EV6. The means of three or more groups were compared upon the presence of one independent variable (genotype) with a one‐way Analysis of Variance (ANOVA) followed by Bonferroni *post hoc* test (analyzed with OriginPro 2020). Where applicable, a Kruskal–Wallis test with Bonferroni *post hoc* test was performed as non‐parametric alternative for a one‐way ANOVA (IBM SPSS statistics 26). Upon the presence of two independent variables (genotype and treatment), a two‐way ANOVA followed by a Bonferroni *post hoc* test was conducted with OriginPro 2020. To analyze the differences across two or more different groups in time‐dependent experiments, a two‐way ANOVA mixed design was conducted with genotype or media as between‐subject and time as within‐subject factor, followed by a Bonferroni *post hoc* test (Origin Pro 2020).

Significances for analyses with one independent variable are indicated with asterisks (***P < 0.001, **P < 0.01, *P < 0.05, ^n.s.^ *P* > 0.05), and for two independent variables main effects are displayed with diamonds (^###^ *P* < 0.001, ^##^ *P* < 0.01, ^#^ *P* < 0.05, ^n.s.^ *P* > 0.05) and simple main effects are depicted as asterisks (***P < 0.001, **P < 0.01, *P < 0.05, ^n.s.^ *P* > 0.05). Calculated *P*‐values are presented in Table EV6. Figures were created with OriginPro 2020 and further processed with CorelDRAW X8.

## Author contributions

Conceptualization: MO, SB, and FF; Methodology: JB, AK, AB, FF, and MO; Validation: FF and MO; Formal Analysis: JB, AK, and SR; Investigation: JB, AK, SR, LM‐B, HD, JD, VK, and FF; Resources: AB, SB, FF, and MO; Data Curation: JB, AK, SR, FF, and MO; Writing—Original Draft: JB, AK, and MO; Writing—Review and Editing: JB, AK, HD, VK, AB, SB, FF, and MO; Visualization: JB, AK, and MO; Supervision: SB, FF, and MO; Project Administration: MO; Funding Acquisition: AK, VK, AB, SB, FF, and MO.

## Conflict of interest

The authors declare that they have no conflict of interest.

## Supporting information



Expanded View Figures PDF

Table EV1

Table EV2

Table EV3

Table EV4

Table EV5

Table EV6

## Data Availability

Cryo‐EM maps and atomic coordinates have been deposited at the Electron Microscopy Data Bank (EMD) and the Protein Data Bank (PDB) with the accession codes for the CIII_2_/CIV [EMD‐10847 (http://www.ebi.ac.uk/pdbe/entry/EMD-10847) and 6YMX (http://www.rcsb.org/pdb/explore/explore.do?structureId=6YMX)] and CIV model [EMD‐10848 (http://www.ebi.ac.uk/pdbe/entry/EMD-10848) and 6YMY (http://www.rcsb.org/pdb/explore/explore.do?structureId=6YMY)].

## Footnotes

1 Correction added on 8 December 2020, after first online publication: The last paragraph of the introduction and the references therein were added. This replaces the previous last two sentences of the introduction.
