# Supplementary material for: Respiratory supercomplexes enhance electron transport by decreasing cytochrome c diffusion distance
Source: EMBO Rep. 2020 Oct 5;21(12):e51015. doi: 10.15252/embr.202051015 (PMC7726804; doi:10.15252/embr.202051015)
Supplement: Supplementary file 1 — Expanded View Figures PDF [file EMBR-21-e51015-s003.pdf]

Expanded View Figures

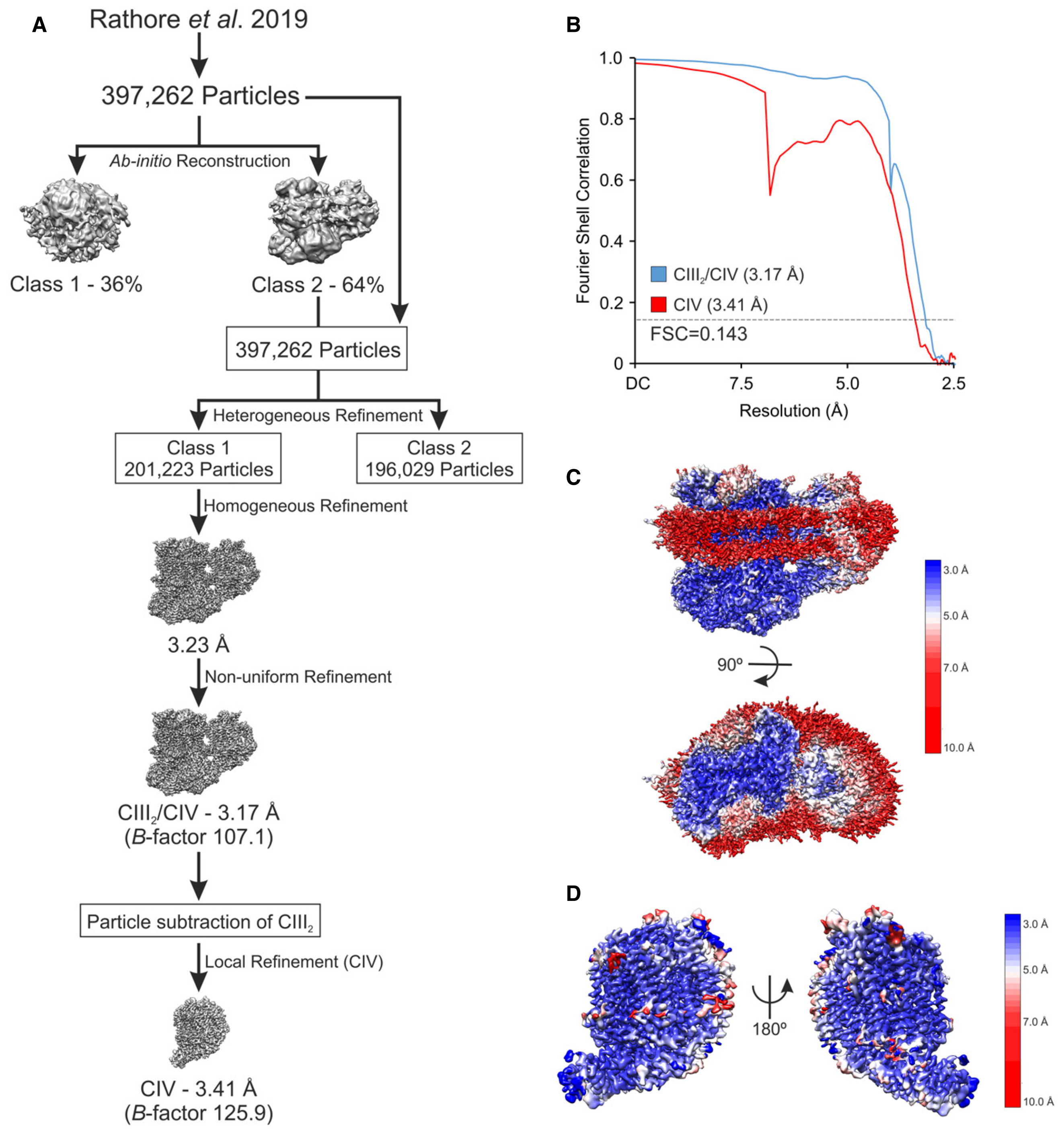

Figure EV1.

© 2020 The Authors

◀ **Figure EV1. Workflow of cryo-EM processing.**

A Schematic illustration of Workflow of cryo-EM processing.

B Gold-standard Fourier shell correlation (FSC) of the $CIII_2$/CIV supercomplex and the CIV density maps after final refinements. FSC = 0.143 is presented as dashed line.

C, D Local resolution maps of the $CIII_2$/CIV supercomplex viewed along the mitochondrial inner membrane (IM) and from the mitochondrial intermembrane space (IMS) side (C), and the CIV density after particle subtraction of $CIII_2$ and the digitonin micelle viewed from two rotations along the mitochondrial inner membrane (D).

© 2020 The Authors

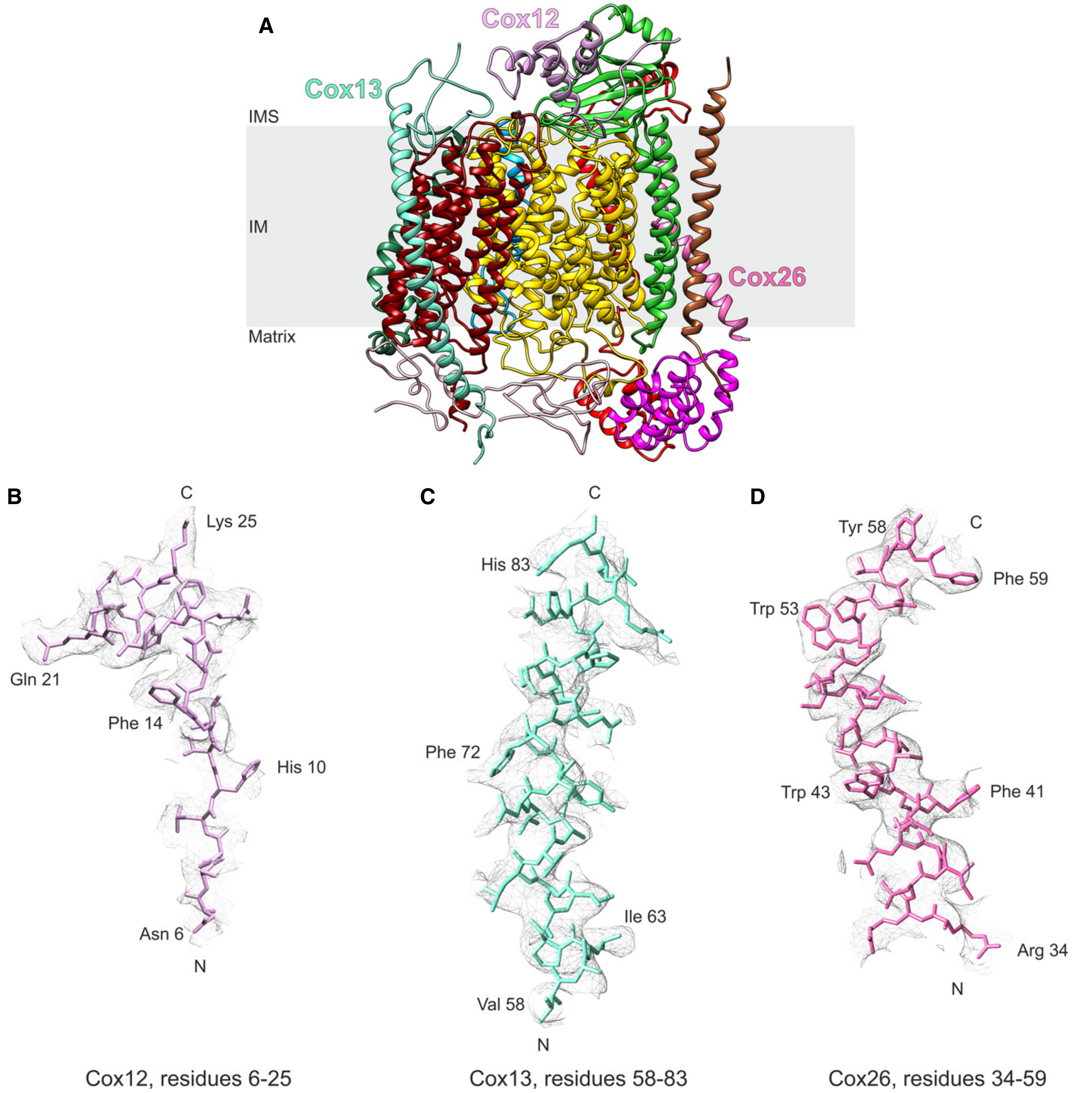

Figure EV2. Example densities of CIV subunits Cox12, Cox13, and Cox26.

A Ribbon diagram of CIV with Cox12, Cox13, and Cox26 annotated. IM: inner membrane; IMS: intermembrane space.

B–D Example densities of Cox12 (B), Cox13 (C), and Cox26 (D) demonstrating parts of the subunits that improved due to the re-processing of the data set.

 © 2020 The Authors

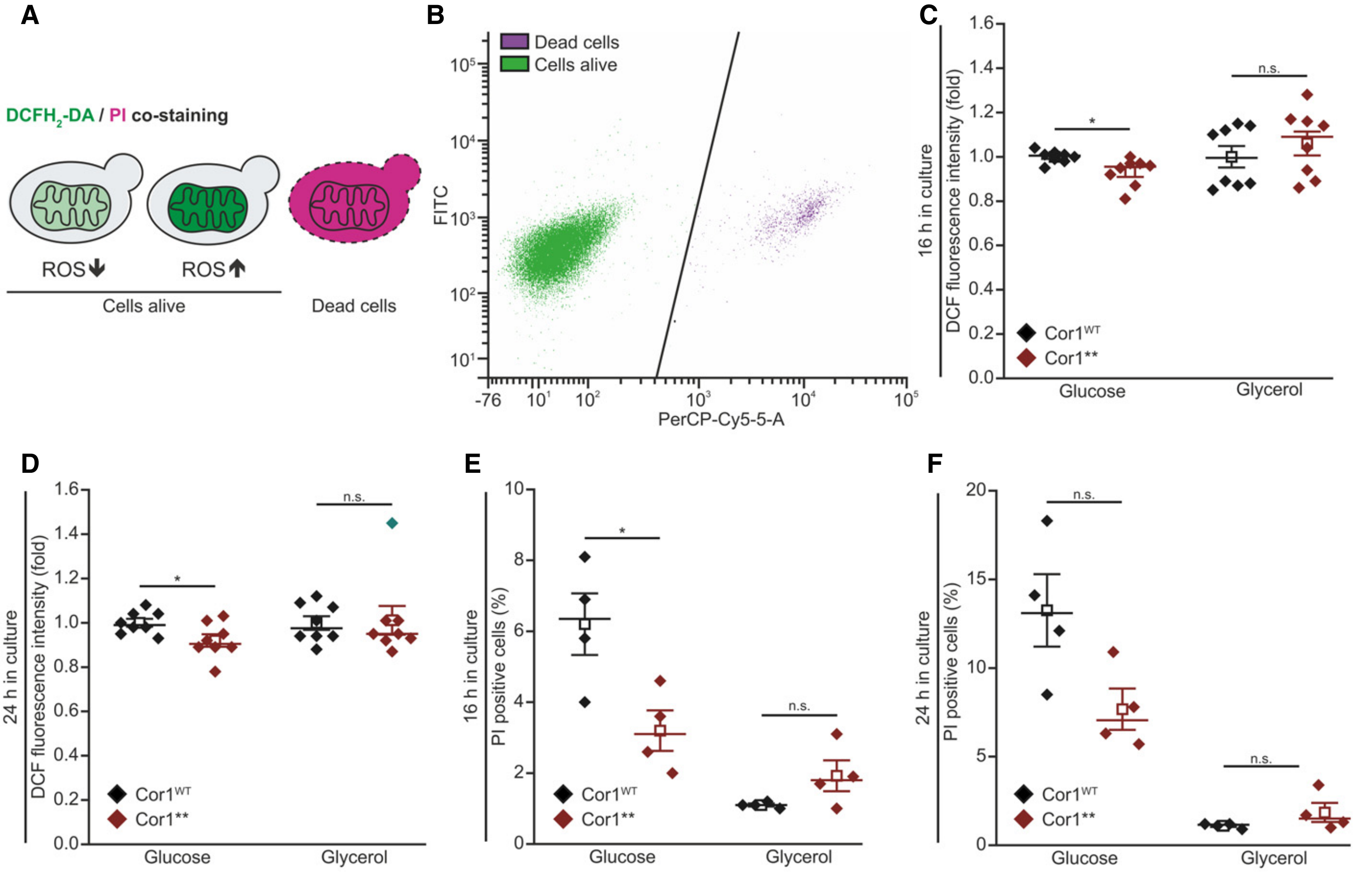

Figure EV3. Disruption of supercomplexes does not modulate oxidative stress or cell death.

A, B Scheme of 2′,7′-dichlorodihydrofluorescein diacetate ($DCFH_2$-DA), and propidium iodide (PI) staining and gating strategy for flow cytometry. PI is taken up by cells upon loss of membrane integrity, thus indicating dead cells. Non-fluorescent $DCFH_2$-DA is oxidized to fluorescent 2′,7′-dichlorofluorescein (DCF) upon oxidative stress (A). Hence, the DCF mean fluorescence intensity of PI-negative cells was applied as a measure for oxidative stress. The threshold used to separate PI-negative and PI-positive cells is shown as black line in (B).

C, D Flow cytometric quantification of oxidative stress indicated via $DCFH_2$-DA/PI counterstaining of cells expressing the wild-type form of Cor1 ($Cor1^{WT}$), as well as the mutant $Cor1^{N63A,\ N187A,\ D192A,\ V189A,\ Y65A,\ L238A,\ K240A}$ (Cor1**). Cells were analyzed 16 h (C) and 24 h (D) after inoculation in media either containing glucose or galactose as indicated. To visualize fold values, normalization was performed to $Cor1^{WT}$ cells of the respective medium used.

E, F Analysis of cell death via flow cytometric quantification of PI stained cells as described above. Experiments were performed 16 h (E) and 24 h (F) after inoculation, and the percentages of PI-positive cells are presented.

Data information: Mean (square) ± s.e.m., median (center line), and single data points are depicted (for C and D: $n = 8$ biological replicates; for E and F: $n = 4$ biological replicates). Outliers were detected using the 2.2-fold interquartile range labeling rule and are indicated in turquoise. Two-tailed independent sample *t*-tests were used for statistical analysis, except for (C glycerol) and (D glycerol), where two-tailed Mann–Whitney *U* tests were applied. Significances are visualized as: n.s.: not significant ($P \geq 0.05$), $*P < 0.05$. A detailed description of statistical analyses performed is given in Table EV6.

© 2020 The Authors

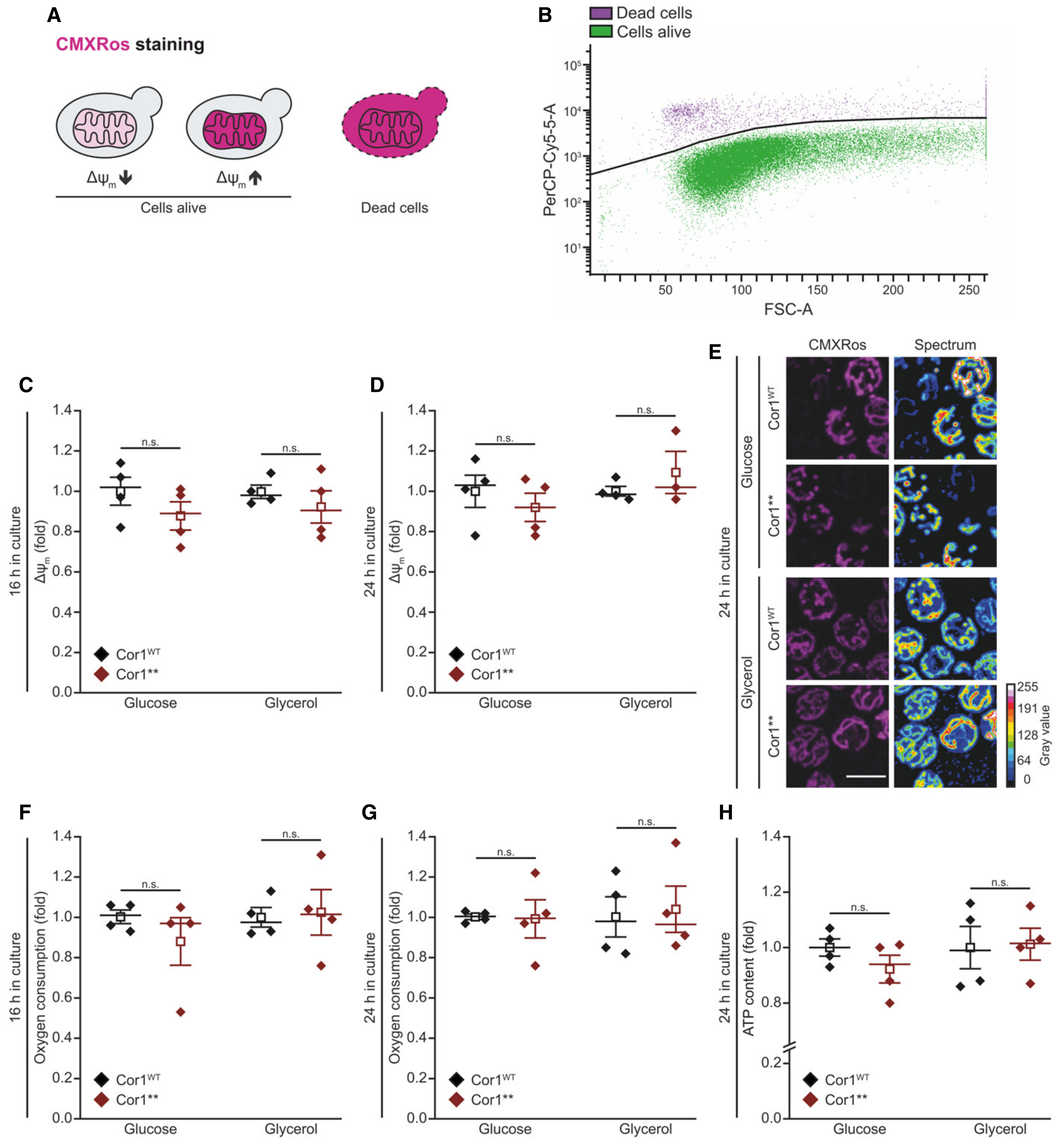

Figure EV4.

© 2020 The Authors

Figure EV4. Absence of supercomplex formation does not impact mitochondrial function in living cells.

A, B Scheme of Mitotracker CMXRos staining and gating strategy for flow cytometric analysis. The fluorescence probe is taken up by mitochondria depending on the transmembrane potential ($\Delta\psi_m$). Dead cells accumulate the dye due to a lack of membrane integrity (A) and are excluded from the analysis. The threshold used to separate dead and living cells is shown as black line in (B). The mean fluorescence intensity of cells alive is quantified as a readout for $\Delta\psi_m$.

C–E Analysis of $\Delta\psi_m$ via Mitotracker CMXRos staining of cells expressing the wild-type form of Cor1 ($Cor1^{WT}$), as well as the mutant $Cor1^{N63A,\ N187A,\ D192A,\ V189A,\ Y65A,\ L238A,\ K240A}$ (Cor1**). Cells were cultivated in CM media containing indicated carbon sources. Flow cytometric quantification 16 h (C) and 24 h (D) after inoculation are shown, as well as representative confocal micrographs (Z-projections) after 24 h (E). Normalization in (C) and (D) was performed to $Cor1^{WT}$ cells of the respective medium used.

F, G Oxygen consumption quantified in intact cells. Strains described in (C) were analyzed 16 h (F) and 24 h (G) after inoculation. Normalization of data was performed as stated in (C) to present fold values.

H Cellular ATP content measured from cells described in (C). The measurement was performed with cells cultivated for 24 h in CM media containing the indicated carbon sources. Normalization was performed as stated in (C).

Data information: Mean (square) $\pm$ s.e.m., median (center line), and single data points ($n = 4$ biological replicates) are depicted. Two-tailed independent sample t-tests were used for statistical analysis. For (C glycerol), Welch correction was performed. Significances are presented as: n.s.: not significant ($P \geq 0.05$). A detailed description of statistical analyses performed is given in Table EV6. Scale bar in (E) represents 5 µm.

© 2020 The Authors

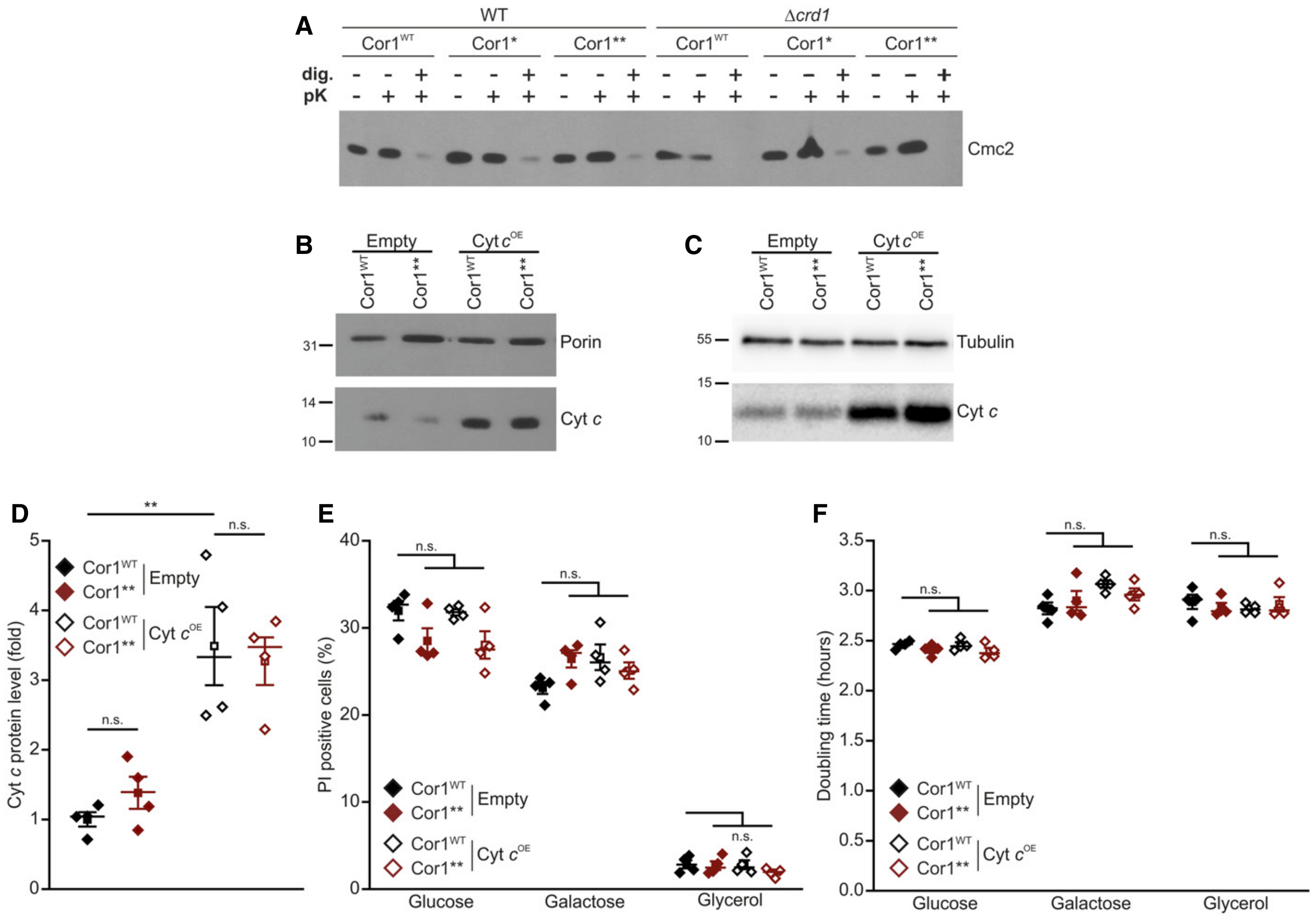

Figure EV5. The lack of supercomplexes impairs electron transport from CIII to CIV.

A Assays to verify the intactness of the outer mitochondrial membrane. Mitochondria were isolated from strains expressing the wild-type form of Cor1 ($Cor1^{WT}$), as well as the mutants $Cor1^{N63A, N187A, D192A}$ (Cor1*) and $Cor1^{N63A, N187A, D192A, V189A, Y65A, L238A, K240A}$ (Cor1**) in wild-type (WT) background as well as in cells lacking *CRD1* (*crd1*Δ). Intactness of the outer mitochondrial membrane was tested by following the stability of the intermembrane protein Cmc2 1 h after treatment with 6 μg/ml proteinase K (pK). Negative control samples were created by membrane permeabilization with 0.5% digitonin (dig.).

B Immunoblot analysis of isolated mitochondria from strains expressing the wild-type form of Cor1 ($Cor1^{WT}$) or the mutant $Cor1^{N63A, N187A, D192A, V189A, Y65A, L238A, K240A}$ (Cor1**). Cells either overexpressed cytochrome *c* (Cyt c^{OE}) or contained the empty plasmid (Empty) as a control. Blots were probed with antibodies against Cyt *c*, as well as porin as loading control.

C Immunoblot analysis as described in (B) from intact cells. Blots were probed with antibodies against Cyt *c*, as well as tubulin as loading control.

D Densitometric quantification of immunoblots validating overexpression of cytochrome *c* (Cyt c^{OE}) in strains expressing either the wild-type form of Cor1 ($Cor1^{WT}$) or the mutant $Cor1^{N63A, N187A, D192A, V189A, Y65A, L238A, K240A}$ (Cor1**). The Cyt *c* signal was normalized to tubulin intensities as a loading control, followed by normalization to $Cor1^{WT}$ strains harboring the empty vector to present fold values.

E Flow cytometric quantification of loss of membrane integrity as visualized via propidium iodide (PI) staining of cells described in (D). Cells were cultivated in CM media either containing glucose, galactose or glycerol as carbon source.

F Doubling time of strains described in (E).

Data information: Mean (square) ± s.e.m., median (center line), and single data points ($n = 4$ biological replicates) are depicted one-way ANOVA followed by Bonferroni *post hoc* test was applied for statistical analysis, and significances are presented as: n.s.: not significant ($P \geq 0.05$); $^{**}P < 0.01$. A detailed description of statistical analyses performed is given in Table EV6.

© 2020 The Authors
