# Supplementary material for: Respiratory supercomplexes enhance electron transport by decreasing cytochrome c diffusion distance
Source: EMBO Rep. 2020 Oct 5;21(12):e51015. doi: 10.15252/embr.202051015 (PMC7726804; doi:10.15252/embr.202051015)
Supplement: Supplementary file 2 — Table EV1 [file EMBR-21-e51015-s002.docx]

Table EV1. Cryo-EM data collection, refinements and validation statistics

|  |  | **CIII/CIV** | **CIV** |
| --- | --- | --- | --- |
| **Data collection** | Microscope | FEI Titan-Krios | FEI Titan-Krios |
|  | Electron source | FEG | FEG |
|  | Voltage (kV) | 300 | 300 |
|  | Electron detector | Gatan K2-summit | Gatan K2-summit |
|  | Micrographs | 8,775 | 8,775 |
|  | Frames/micrograph | 40 | 40 |
|  | Magnification (nominal) | 130,000 | 130,000 |
|  | Magnification (calibrated) | 47,170 | 47,170 |
|  | Pixel size (Å) | 1.06 | 1.06 |
|  | Defocus range (µm) | 1.4 – 3.0 | 1.4 – 3.0 |
|  | Electron dose (eÅ^-2^) | 40 | 40 |
|  | Electron/camera pixel (e/Å^2^/s) | 4.394 | 4.394 |
| **3D-reconstruction** | Number of particles | 201,223 | 201,223 |
|  | Resolution (Å) (FSC=0.143) | 3.17 | 3.41 |
|  | Map sharpening B-factor (Å^2^) | 107.1 | 125.9 |
| **Model composition** | Non-hydrogen atoms | 47034 | 14612 |
|  | Protein residues | 5759 | 1766 |
|  | Ligands | 38 | 16 |
| **Model validation** | CC (mask) Model vs. Data  CC (box) Model vs. Data  CC (peaks) Model vs. Data  CC (volume) Model vs. Data  Mean CC for ligands  Ramachandran Favoured (%)  Ramachandran Outliers (%)  Ramachandran Allowed (%) | 0.85  0.76  0.69  0.84  0.69  93.89  0.05  6.06 | 0.81  0.68  0.59  0.82  0.69  91.04  0.06  8.90 |
|  | RMSD Bond lengths (Å)  RMSD Bond angles (°)  Clashscore, all atoms  Rotamer outliers (%)  Molprobity score | 0.013  0.960  6.91  0.39  1.79 | 0.010  0.937  7.43  0.27  1.93 |
