# Supplementary material for: Respiratory supercomplexes enhance electron transport by decreasing cytochrome c diffusion distance
Source: EMBO Rep. 2020 Oct 5;21(12):e51015. doi: 10.15252/embr.202051015 (PMC7726804; doi:10.15252/embr.202051015)
Supplement: Supplementary file 3 — Table EV2 [file EMBR-21-e51015-s001.docx]

Table EV2: Calculated heme concentrations from spectra depicted in Figure 2 B. For each sample 200 µg of mitochondria were applied for the measurement.

|  | **Heme concentration (µM)** | | | | **Heme ratios** | |
| --- | --- | --- | --- | --- | --- | --- |
| **Strain** | **[*cc*_1_]** | **[*c*]** | **[*b*]** | **[*aa_3_*]** | **[c*c*_1_]/ [*b*]** | **[*b*]/ [*aa*_3_]** |
| Cor1^WT^ | 2.08 ± 0.01 | 0.88 ± 0.08 | 0.97 ± 0.05 | 0.69 ± 0.03 | 2.12 | 1.41 |
| Cor1^*^ | 2.69 ± 0.16 | 1.21 ± 0.07 | 0.71 ± 0.10 | 0.91 ± 0.04 | 3.81 | 0.78 |
| Cor1^**^ | 1.96 ± 0.16 | 1.01 ± 0.10 | 0.87 ± 0.06 | 0.78 ± 0.09 | 2.25 | 1.13 |
| *crd1*Δ Cor1^WT^ | 1.95 ± 0.06 | 1.21 ± 0.06 | 0.85 ± 0.04 | 0.73 ± 0.09 | 2.30 | 1.17 |
| *crd1*Δ Cor1^*^ | 2.64 ± 0.06 | 1.79 ± 0.03 | 0.54 ± 0.06 | 1.04 ± 0.11 | 4.94 | 0.52 |
| *crd1*Δ Cor1^**^ | 2.02 ± 0.03 | 1.24 ± 0.08 | 0.84 ± 0.13 | 0.75 ± 0.09 | 2.41 | 1.12 |
