# Supplementary material for: Respiratory supercomplexes enhance electron transport by decreasing cytochrome c diffusion distance
Source: EMBO Rep. 2020 Oct 5;21(12):e51015. doi: 10.15252/embr.202051015 (PMC7726804; doi:10.15252/embr.202051015)
Supplement: Supplementary file 4 — Table EV3 [file EMBR-21-e51015-s007.docx]

Table EV3: Strains used in this study

| **Strain and genotype** | **Source** |
| --- | --- |
| *S. cerevisiae*: Strain MOY119: W303, *Mat* **α** *ura3-1; trp1-1, leu2-3,112; his3-11,15; ade2-1; can1-100; arg8::HIS3;* ρ^+^; wt; intronless | (Gruschke *et al.*, 2011) |
| *S. cerevisiae*: Strain MOY1460: W303, *Mat* **α** *ura3-1; trp1-1, leu2-3,112; his3-11,15; ade2-1; can1-100; arg8::HIS3; cor1::kanMX4;* pRS305*COR1 wild type;* ρ^+^; wt; intronless | This study |
| *S. cerevisiae*: Strain MOY1461: W303, *Mat* **α** *ura3-1; trp1-1, leu2-3,112; his3-11,15; ade2-1; can1-100; arg8::HIS3; cor1::kanMX4;* pRS305*COR1-Y237D; rho^+^ type;* ρ^+^; wt; intronless | This study |
| *S. cerevisiae*: Strain MOY1462: W303, *Mat* **α** *ura3-1; trp1-1, leu2-3,112; his3-11,15; ade2-1; can1-100; arg8::HIS3; cor1::kanMX4;* pRS305*COR1-K240D type;* ρ^+^; wt; intronless | This study |
| *S. cerevisiae*: Strain MOY1463: W303, *Mat* **α** *ura3-1; trp1-1, leu2-3,112; his3-11,15; ade2-1; can1-100; arg8::HIS3; cor1::kanMX4;* pRS305*COR1-N187A type;* ρ^+^; wt; intronless | This study |
| *S. cerevisiae*: Strain MOY1465: W303, *Mat* **α** *ura3-1; trp1-1, leu2-3,112; his3-11,15; ade2-1; can1-100; arg8::HIS3; cor1::kanMX4;* pRS305*COR1-N63A-N187A-D192A type;* ρ^+^; wt; intronless | This study |
| *S. cerevisiae*: Strain MOY1466: W303, *Mat* **α** *ura3-1; trp1-1, leu2-3,112; his3-11,15; ade2-1; can1-100; arg8::HIS3; cor1::kanMX4;* pRS305*COR1-K240D-L241A-Y65A type;* ρ^+^; wt; intronless | This study |
| *S. cerevisiae*: Strain MOY1467: W303, *Mat* **α** *ura3-1; trp1-1, leu2-3,112; his3-11,15; ade2-1; can1-100; arg8::HIS3; cor1::kanMX4;* pRS305*COR1-V237A-L238A-N199A type;* ρ^+^; wt; intronless | This study |
| *S. cerevisiae*: Strain MOY1468: W303, *Mat* **α** *ura3-1; trp1-1, leu2-3,112; his3-11,15; ade2-1; can1-100; arg8::HIS3; cor1::kanMX4;* pRS305*COR1-Y65S-N187A type;* ρ^+^; wt; intronless | This study |
| *S. cerevisiae*: Strain MOY1469: W303, *Mat* **α** *ura3-1; trp1-1, leu2-3,112; his3-11,15; ade2-1; can1-100; arg8::HIS3; cor1::kanMX4;* pRS305*COR1-Y65S-K240D type;* ρ^+^; wt; intronless | This study |
| *S. cerevisiae*: Strain MOY1487: W303, *Mat* **α** *ura3-1; trp1-1, leu2-3,112; his3-11,15; ade2-1; can1-100; arg8::HIS3; cor1::kanMX4;* pRS305*COR1-N63A-Y65A-N187A-V189A-D192A-L238A-K240A type;* ρ^+^; wt; intronless | This study |
| *S. cerevisiae*: Strain MOY1500: W303, *Mat* **α** *ura3-1; trp1-1, leu2-3,112; his3-11,15; ade2-1; can1-100; arg8::HIS3; cor1::kanMX4; crd1::TRP1;* pRS305*COR1 wild type type;* ρ^+^; wt; intronless | This study |
| *S. cerevisiae*: Strain MOY1501: W303, *Mat* **α** *ura3-1; trp1-1, leu2-3,112; his3-11,15; ade2-1; can1-100; arg8::HIS3; cor1::kanMX4; crd1::TRP1;* pRS305*COR1-N63A-N187A-D192A type;* ρ^+^; wt; intronless | This study |
| *S. cerevisiae*: Strain MOY1502: W303, *Mat* **α** *ura3-1; trp1-1, leu2-3,112; his3-11,15; ade2-1; can1-100; arg8::HIS3; cor1::kanMX4; crd1::TRP1;* pRS305*COR1-N63A-Y65A-N187A-V189A-D192A-L238A-K240A type;* ρ^+^; wt; intronless | This study |
