# Supplementary material for: Respiratory supercomplexes enhance electron transport by decreasing cytochrome c diffusion distance
Source: EMBO Rep. 2020 Oct 5;21(12):e51015. doi: 10.15252/embr.202051015 (PMC7726804; doi:10.15252/embr.202051015)
Supplement: Supplementary file 5 — Table EV4 [file EMBR-21-e51015-s006.docx]

Table EV4: Oligonucleotides used in this study

| **Oligonucleotide** | **Source** |
| --- | --- |
| *COR*1_fw: 5’- CAGAAGTTCAAGGGCCCGCGCTGTC -3’ | Sigma-Aldrich |
| *COR*1_rev: 5’- GATCGAGAGATGACTTCTCTACCAAACAACTTCC -3’ | Sigma-Aldrich |
| *CRD*1_fw: 5’- GGAGTGTGAAAAAGCTCGATTCATGAAGCCATCCTCTTA  AAGGAAGCATTTAATAGACAGCATCG -3‘ | Sigma-Aldrich |
| *CRD*1_rev: 5’- TGATTTAGTGTATTTTCCACTACATAAAAACTAAAAAACC  TGATGCGGTATTTTCTCCTTACGC -3‘ | Sigma-Aldrich |
| *HIS3*_fw: 5’- GATTTCTTCGAAGAATATACTAAAAAATG -3’ | Eurofins Genomics |
| *HIS3*_rev: 5’- ATACTTACTGACATTCATAGGTATAC -3’ | Eurofins Genomics |
| *CYC*1_fw: 5’- ATGACTGAATTCAAGGCCGGTTC -3’ | Sigma-Aldrich |
| *CYC*1_rev: 5’- TTACTCACAGGCTTTTTTCAAGTAGGTAATTAAG -3’ | Sigma-Aldrich |
| pCM190_fw: 5’- CTACTTGAAAAAAGCCTGTGAGTAAGAGGGCCGCATCAT  GTAATTAGTTATG -3’ | Sigma-Aldrich |
| pCM190_rev: 5’- CAGAACCGGCCTTGAATTCAGTCATGGTGGCGGCGGGCCCGAATTGATCC  GGTAATTTAGTG -3’ | Sigma-Aldrich |
