# Supplementary material for: Respiratory supercomplexes enhance electron transport by decreasing cytochrome c diffusion distance
Source: EMBO Rep. 2020 Oct 5;21(12):e51015. doi: 10.15252/embr.202051015 (PMC7726804; doi:10.15252/embr.202051015)
Supplement: Supplementary file 6 — Table EV5 [file EMBR-21-e51015-s005.docx]

Table EV5: Antibodies used in this study

| **Antibodies** | **Source** |
| --- | --- |
| Anti-Cox1 | Abcam; ab110270 |
| Anti-Cox5 | (Liu and Barrientos, 2013) |
| Anti-Cox12 | Gift from Dr. J. Brix |
| Anti-Cox13 | Gift from Dr. J. Brix |
| Anti-Cyt*b* | Gift from Dr. A. Tzagoloff |
| Anti-Rip1 | Gift from Dr. R. Stuart |
| Anti-Cyt *c* | (Barrientos *et al.*, 2003) |
| Anti-Rcf1 | Gift from Dr. R. Stuart |
| Anti-Cmc2 | (Horn, Fontanesi and Barrientos, 2008) |
| Anti-Porin | Abcam; ab110326 |
| Anti-Tom70 | (Hildenbeutel *et al.*, 2014) |
| Anti-Cor1 | (Hildenbeutel *et al.*, 2014) |
| Anti-Qcr7 | (Gruschke *et al.*, 2012) |
| Anti rabbit | BioRad; 1705046 |
| Anti tubulin | Abcam; ab184970 |
