# Supplementary material for: Respiratory supercomplexes enhance electron transport by decreasing cytochrome c diffusion distance
Source: EMBO Rep. 2020 Oct 5;21(12):e51015. doi: 10.15252/embr.202051015 (PMC7726804; doi:10.15252/embr.202051015)
Supplement: Supplementary file 7 — Table EV6 [file EMBR-21-e51015-s008.docx]

Table EV6: Detailed description of statistical analyses performed in this study.

Comparisons with p-values below 0.05 are marked in bold.

| **Analyzed data** | **Statistical test** | **Additional information** | **Result** |
| --- | --- | --- | --- |
| **Figure 3 A** |  |  |  |
| WT on glucose | Two-tailed independent-sample t-test | All assumptions met | Cor1^WT^ vs. Cor1^**^:  t(6)=0.392, p=0.708 |
| WT on glycerol | Two-tailed independent-sample t-test | All assumptions met | Cor1^WT^ vs. Cor1^**^:  t(6)=0.900, p=0.403 |
| **Figure 3 B** |  |  |  |
| Cultivated on glucose | Two-Way ANOVA mixed design with Bonferroni post hoc test | Cor1-variant was considered as between-subject factor, time as within-subject factor; sphericity confirmed via Greenhouse-Geisser correction; significant interaction between Cor1-variant and time detected;  test conducted despite significantly different variances, due to identical sample size (n=4); Significances of main effects visualized with # | Main effects for Cor1-variant:  F(1,6)=0.022, p = 0.886 |
| Cultivated on glycerol | Two-Way ANOVA mixed design with Bonferroni post hoc test | Cor1-variant was considered as between-subject factor, time as within-subject factor; sphericity confirmed via Greenhouse-Geisser correction; no significant interaction between Cor1-variant and time detected;  test conducted despite significantly different variances, due to identical sample size (n=4); Significances of main effects visualized with # | Main effects for Cor1-variant:  F(1,6)=5.140, p = 0.064 |
| **Figure 3 D** |  |  |  |
| Cultivated on glucose | Two-Way ANOVA mixed design with Bonferroni post hoc test | Cor1-variant was considered as between-subject factor, time as within-subject factor; sphericity confirmed via Greenhouse-Geisser correction; no significant interaction between Cor1-variant and time detected;  Significances of main effects visualized with # | Main effects for Cor1-variant:  F(1,6)=0.479, p = 0.515 |
| Cultivated on galactose | Two-Way ANOVA mixed design with Bonferroni post hoc test | Cor1-variant was considered as between-subject factor, time as within-subject factor; sphericity confirmed via Mauchly’s test; significant interaction between Cor1-variant and time detected;  Significances of main effects visualized with # | Main effects for Cor1-variant:  **F(1,6)=360.553, p = 0.000** |
| Cultivated on glycerol | Two-Way ANOVA mixed design with Bonferroni post hoc test | Cor1-variant was considered as between-subject factor, time as within-subject factor; sphericity confirmed via Mauchly’s test; significant interaction between Cor1-variant and time detected;  Significances of main effects visualized with # | Main effects for Cor1-variant:  **F(1,6)=95.309, p = 0.000** |
| **Figure 3 E** |  |  |  |
| Cultivated on glucose | Two-Way ANOVA mixed design with Bonferroni post hoc test | Cor1-variant was considered as between-subject factor, time as within-subject factor; sphericity confirmed via Mauchly’s test; no significant interaction between Cor1-variant and time detected;  Significances of main effects visualized with # | Main effects for Cor1-variant:  F(1,6)=3.789, p = 0.100 |
| Cultivated on galactose | Two-Way ANOVA mixed design with Bonferroni post hoc test | Cor1-variant was considered as between-subject factor, time as within-subject factor; sphericity confirmed via Greenhouse-Geisser correction; significant interaction between Cor1-variant and time detected;  Significances of main effects visualized with # | Main effects for Cor1-variant:  **F(1,6)=556.690, p = 0.000** |
| Cultivated on glycerol | Two-Way ANOVA mixed design with Bonferroni post hoc test | Cor1-variant was considered as between-subject factor, time as within-subject factor; sphericity confirmed via Mauchly’s test; significant interaction between Cor1-variant and time detected;  Significances of main effects visualized with # | Main effects for Cor1-variant:  **F(1,6)=2106.358, p = 0.000** |
| **Figure 4 A** |  |  |  |
| CIV activity | Two-tailed independent-sample t-test with Welch correction | Significantly different variances detected with Levene’s test (p=0.016); Welch correction was performed | **Cor1^WT^ vs. Cor1^**^:**  **t(6.767)=-16.208, p=0.000** |
| **Figure 4 B** |  |  |  |
| CIII activity | Two-tailed independent-sample t-test with Welch correction | Significantly different variances detected with Levene’s test (p=0.024); Welch correction was performed | **Cor1^WT^ vs. Cor1^**^:**  **t(4.708)=3.564, p=0.018** |
| **Figure 4 C** |  |  |  |
| Basal | Two-tailed independent-sample t-test | All assumptions met | **Cor1^WT^ vs. Cor1^**^:**  **t(4)=8.126, p=0.001** |
| Phosphorylating | Two-tailed independent-sample t-test | All assumptions met | **Cor1^WT^ vs. Cor1^**^:**  **t(4)=13.724, p=0.000** |
| **Figure 4 D** |  |  |  |
| Basal | Two-tailed independent-sample t-test | All assumptions met | **Cor1^WT^ vs. Cor1^**^:**  **t(4)=9.585, p=0.000** |
| Phosphorylating | Two-tailed independent-sample t-test | All assumptions met | **Cor1^WT^ vs. Cor1^**^:**  **t(4)=35.237, p=0.000** |
| **Figure 4 E** |  |  |  |
| NADH | Two-tailed independent-sample t-test | All assumptions met | Cor1^WT^ vs. Cor1^**^:  t(4)=1.214, p=0.292 |
| Succinate+  G3P | Two-tailed Mann-Whitney U test | Non-normally distributed data detected via Shapiro-Wilk test; Mann-Whitney U test performed as an alternative for independent sample t-test | Cor1^WT^ vs. Cor1^**^:  U(*n*≥3)=0, p=0.052 |
| **Figure 4 F** |  |  |  |
| – Cyt *c* | Two-tailed independent-sample t-test with Welch correction | Significantly different variances detected with Levene’s test (p=0.042); Welch correction was performed | **Cor1^WT^ vs. Cor1^**^:**  **t(2.179)=22.428, p=0.001** |
| + Cyt *c* | Two-tailed independent-sample t-test | All assumptions met | Cor1^WT^ vs. Cor1^**^:  t(4)=2.408, p=0.074 |
| **Figure 4 G** |  |  |  |
| Ratio  +Cyt *c/*- Cyt *c* | Two-tailed independent-sample t-test | All assumptions met | **Cor1^WT^ vs. Cor1^**^:**  **t(4)=-17.304, p=0.000** |
| **Figure 5 A** |  |  |  |
| Basal | One-Way ANOVA with Bonferroni post hoc test | All assumptions met | **F(3,23) = 104.526, p = 0.000**  **Cor^WT^ Empty vs.**  **Cor^**^ Empty: p = 0.000**  Cor^WT^ Empty vs.  Cor^WT^ Cyt *c*^OE^: p = 0.159  **Cor^WT^ Cyt *c*^OE^ vs.**  **Cor^**^ Empty: p = 0.000**  **Cor^WT^ Empty vs.**  **Cor^**^ Cyt *c*^OE^: p = 0.000**  **Cor^**^ Empty vs.**  **Cor^**^ Cyt *c*^OE^: p = 0.000**  **Cor^WT^ Cyt *c*^OE^ vs.**  **Cor^**^ Cyt *c*^OE^: p = 0.000** |
| Phosphorylating | One-Way ANOVA with Bonferroni post hoc test | All assumptions met | **F(3,23) = 173.917, p = 0.000**  **Cor^WT^ Empty vs.**  **Cor^**^ Empty: p = 0.000**  Cor^WT^ Empty vs.  Cor^WT^ Cyt *c*^OE^: p = 1.000  **Cor^WT^ Cyt *c*^OE^ vs.**  **Cor^**^ Empty: p = 0.000**  **Cor^WT^ Empty vs.**  **Cor^**^ Cyt *c*^OE^: p = 0.000**  **Cor^**^ Empty vs.**  **Cor^**^ Cyt *c*^OE^: p = 0.000**  **Cor^WT^ Cyt *c*^OE^ vs.**  **Cor^**^ Cyt *c*^OE^: p = 0.000** |
| **Figure 5 B** | Two-Way ANOVA mixed design with Bonferroni post hoc test | Expression type was considered as between-subject factor, time as within-subject factor; sphericity confirmed via Mauchly’s test; no significant interaction between Cor1-variants and time detected;  Significances of simple main effects for expression type visualized with * | Main effects for expression type:  F(2,9)=0.153, p = 0.860  Simple main effects for expression type:  Empty/Empty vs.  Cyt *c*^OE^/ Cyt *c*^OE^: p = 1.000  Empty/Empty vs.  Empty/ Cyt *c*^OE^: p = 1.000  Cyt *c*^OE^/Cyt *c*^OE^.  Empty/ Cyt *c*^OE^: p = 1.000 |
| **Figure 5 C** | Two-Way ANOVA mixed design with Bonferroni post hoc test | Expression type was considered as between-subject factor, time as within-subject factor; sphericity confirmed via Mauchly’s test; no significant interaction between Cor1-variants and time detected;  Significances of simple main effects for expression type visualized with * | Main effects for expression type:  **F(2,9)=13.125, p = 0.002**  Simple main effects for expression type:  **Empty/Empty vs.**  **Cyt *c*^OE^/ Cyt *c*^OE^: p = 0.005**  **Empty/Empty vs.**  **Empty/ Cyt *c*^OE^: p = 0.005**  Cyt *c*^OE^/Cyt *c*^OE^.  Empty/ Cyt *c*^OE^: p = 1.000 |
| **Figure 5 D** | Two-Way ANOVA mixed design with Bonferroni post hoc test | Expression type was considered as between-subject factor, time as within-subject factor; sphericity confirmed via Mauchly’s test; no significant interaction between Cor1-variants and time detected;  Significances of simple main effects for expression type visualized with * | Main effects for expression type:  **F(2,9)=9.893, p = 0.006**  Simple main effects for expression type:  **Empty/Empty vs.**  **Cyt *c*^OE^/ Cyt *c*^OE^: p = 0.011**  **Empty/Empty vs.**  **Empty/ Cyt *c*^OE^: p = 0.012**  Cyt *c*^OE^/Cyt *c*^OE^.  Empty/ Cyt *c*^OE^: p = 1.000 |
| **Figure EV3 C** |  |  |  |
| Cultivated on glucose | Two-tailed independent-sample t-test | All assumptions met | **Cor1^WT^ vs. Cor1^**^:**  **t(14)=2.846, p=0.013** |
| Cultivated on glycerol | Two-tailed Mann-Whitney U test | Non-normally distributed data detected via Shapiro-Wilk test; Mann-Whitney U test performed as an alternative for independent sample t-test | Cor1^WT^ vs. Cor1^**^:  U(*n*=8)=21, p=0.266 |
| **Figure EV3 D** |  |  |  |
| Cultivated on glucose | Two-tailed independent-sample t-test | All assumptions met | **Cor1^WT^ vs. Cor1^**^:**  **t(14)=2.420, p=0.029** |
| Cultivated on glycerol | Two-tailed Mann-Whitney U test | Outlier detected (marked in turquoise); Mann-Whitney U test performed as an alternative for independent sample t-test | Cor1^WT^ vs. Cor1^**^:  U(*n*=8)=37, p=634 |
| **Figure EV3 E** |  |  |  |
| Cultivated on glucose | Two-tailed independent-sample t-test | All assumptions met | **Cor1^WT^ vs. Cor1^**^:**  **t(6)=2.880, p=0.028** |
| Cultivated on glycerol | Two-tailed independent-sample t-test | All assumptions met | Cor1^WT^ vs. Cor1^**^:  t(6)=-1.881, p=0.109 |
| **Figure EV3 F** |  |  |  |
| Cultivated on glucose | Two-tailed independent-sample t-test | All assumptions met | Cor1^WT^ vs. Cor1^**^:  t(6)=-1.397, p=0.325 |
| Cultivated on glycerol | Two-tailed independent-sample t-test | All assumptions met | Cor1^WT^ vs. Cor1^**^:  t(6)=-1.387, p=0.215 |
| **Figure EV4 C** |  |  |  |
| Cultivated on glucose | Two-tailed independent-sample t-test | All assumptions met | Cor1^WT^ vs. Cor1^**^:  t(6)=1.242, p=0.260 |
| Cultivated on glycerol | Two-tailed independent-sample t-test with Welch correction | Significantly different variances detected with Levene’s test; therefore, Welch correction was performed | Cor1^WT^ vs. Cor1^**^:  t(4.004)=0.864, p=0.436 |
| **Figure EV4 D** |  |  |  |
| Cultivated on glucose | Two-tailed independent-sample t-test | All assumptions met | Cor1^WT^ vs. Cor1^**^:  t(6)=0.752, p=0.480 |
| Cultivated on glycerol | Two-tailed independent-sample t-test | All assumptions met | Cor1^WT^ vs. Cor1^**^:  t(6)=-0.860, p=0.422 |
| **Figure EV4 F** |  |  |  |
| Cultivated on glucose | Two-tailed independent-sample t-test | All assumptions met | Cor1^WT^ vs. Cor1^**^:  t(6)=0.997, p=0.357 |
| Cultivated on glycerol | Two-tailed independent-sample t-test | All assumptions met | Cor1^WT^ vs. Cor1^**^:  t(6)=-0.203, p=0.847 |
| **Figure EV4 G** |  |  |  |
| Cultivated on glucose | Two-tailed independent-sample t-test | All assumptions met | Cor1^WT^ vs. Cor1^**^:  t(6)=0.1047, p=0.920 |
| **Figure EV4 H** |  |  |  |
| Cultivated on glucose | Two-tailed independent-sample t-test | All assumptions met | Cor1^WT^ vs. Cor1^**^:  t(6)=1.309, p=0.238 |
| Cultivated on glycerol | Two-tailed independent-sample t-test | All assumptions met | Cor1^WT^ vs. Cor1^**^:  t(6)=-0.131, p=0.900 |
| **Figure EV5 D** | One-Way ANOVA with Bonferroni post hoc test | All assumptions met | **F(3,12) = 13.122, p = 0.000**  Cor^WT^ Empty vs.  Cor^**^ Empty: p = 1.000  **Cor^WT^ Empty vs.**  **Cor^WT^ Cyt *c*^OE^: p = 0.002**  **Cor^WT^ Cyt *c*^OE^ vs.**  **Cor^**^ Empty: p = 0.007**  **Cor^WT^ Empty vs.**  **Cor^**^ Cyt *c*^OE^: p = 0.004**  **Cor^**^ Empty vs.**  **Cor^**^ Cyt *c*^OE^: p = 0.015**  Cor^WT^ Cyt *c*^OE^ vs.  Cor^**^ Cyt *c*^OE^: p = 1.000 |
| **Figure EV5 E** |  |  |  |
| Cultivated on glucose | One-Way ANOVA with Bonferroni post hoc test | All assumptions met | F(3,12) = 2.935, p = 0.076 |
| Cultivated on galactose | One-Way ANOVA with Bonferroni post hoc test | All assumptions met | F(3,12) = 2.349, p = 0.124 |
| Cultivated on glycerol | One-Way ANOVA with Bonferroni post hoc test | All assumptions met | F(3,12) = 1.028, p = 0.415 |
| **Figure EV5 F** |  |  |  |
| Cultivated on glucose | One-Way ANOVA with Bonferroni post hoc test | All assumptions met | F(3,12) = 1.404, p = 0.290 |
| Cultivated on galactose | One-Way ANOVA with Bonferroni post hoc test | All assumptions met | F(3,12) = 2.372, p = 0.122 |
| Cultivated on glycerol | One-Way ANOVA with Bonferroni post hoc test | All assumptions met | F(3,12) = 0.287, p = 0.835 |
