# Supplementary material for: Respiratory supercomplexes enhance electron transport by decreasing cytochrome c diffusion distance
Source: EMBO Rep. 2020 Oct 5;21(12):e51015. doi: 10.15252/embr.202051015 (PMC7726804; doi:10.15252/embr.202051015)
Supplement: Supplementary file 8 [file EMBR-21-e51015-s004.pdf]

EMBO reports

Respiratory supercomplexes enhance electron transport by decreasing cytochrome *c* diffusion distance

Jens Berndtsson, Andreas Aufschnaiter, Sorbhi Rathore, Lorena Marin-Buera, Hannah Dawitz, Jutta Diessl, Verena Kohler, Antoni Barrientos, Sabrina Büttner, Flavia Fontanesi, and Martin Ott
DOI: 10.15252/embr.202051015

Corresponding author(s): Martin Ott (martin.ott@dbb.su.se) , Flavia Fontanesi (ffontanesi@med.miami.edu), Sabrina Büttner (sabrina.buettner@su.se)

Review Timeline:

Submission Date:	2nd Jun 20
Editorial Decision:	30th Jun 20
Revision Received:	12th Aug 20
Editorial Decision:	31st Aug 20
Revision Received:	2nd Sep 20
Accepted:	10th Sep 20

Editor: Deniz Senyilmaz Tiebe

Transaction Report:

(Note: With the exception of the correction of typographical or spelling errors that could be a source of ambiguity, letters and reports are not edited. Depending on transfer agreements, referee reports obtained elsewhere may or may not be included in this compilation. Referee reports are anonymous unless the Referee chooses to sign their reports.)

1st Editorial Decision 30th Jun 2020

Dear Prof. Ott,

Thank you for the submission of your research manuscript to our journal, which was now seen by three referees, whose reports are copied below.

We concur with the referees that the presented structural analysis and the findings revealing the functional role of supercomplex are very interesting. However, the minor concerns raised by the referees need to be addressed for publication here.

I find the reports informed and constructive, and believe that addressing the concerns raised will significantly strengthen the manuscript. Considering the amount of work required to address these concerns, we believe that three weeks should be sufficient to revise the manuscript. Please let me know if you anticipate problems meeting this deadline.

When addressing the 1st point of referee #1, please keep in mind that our limit for titles is 100 characters (including spaces).

Given these positive requirements, we would like to invite you to revise your manuscript with the understanding that the referee concerns (as in their reports) must be fully addressed and their suggestions taken on board. Please address all referee concerns in a complete point-by-point response. Acceptance of the manuscript will depend on a positive outcome of a second round of review. It is EMBO reports policy to allow a single round of revision only and acceptance or rejection of the manuscript will therefore depend on the completeness of your responses included in the next, final version of the manuscript.

As a matter of policy, competing manuscripts published during this period will not negatively impact on our assessment of the conceptual advance presented by your study. However, we request that you contact the editor as soon as possible upon publication of any related work, to discuss how to proceed.

*** Temporary update to EMBO Press scooping protection policy:
We are aware that many laboratories cannot function at full efficiency during the current COVID-19/SARS-CoV-2 pandemic and have therefore extended our 'scooping protection policy' to cover the period required for a full revision to address the experimental issues highlighted in the editorial decision letter. Please contact the scientific editor handling your manuscript to discuss a revision plan should you need additional time, and also if you see a paper with related content published elsewhere.***

IMPORTANT NOTE: we perform an initial quality control of all revised manuscripts before re-review. Your manuscript will FAIL this control and the handling will be DELAYED if the following APPLIES:
1. A data availability section providing access to data deposited in public databases is missing (where applicable).
2. Your manuscript contains statistics and error bars based on n=2 or on technical replicates. Please use scatter plots in these cases.

Supplementary/additional data: The Expanded View format, which will be displayed in the main HTML of the paper in a collapsible format, has replaced the Supplementary information. You can submit up to 5 images as Expanded View. Please follow the nomenclature Figure EV1, Figure EV2

etc. The figure legend for these should be included in the main manuscript document file in a section called Expanded View Figure Legends after the main Figure Legends section. Additional Supplementary material should be supplied as a single pdf labeled Appendix. The Appendix includes a table of content on the first page with page numbers, all figures and their legends. Please follow the nomenclature Appendix Figure Sx throughout the text and also label the figures according to this nomenclature. For more details please refer to our guide to authors.

Please note that for all articles published beginning 1 July 2020, the EMBO Reports reference style will change to the Harvard style for all article types. Details and examples are provided at https://www.embopress.org/page/journal/14693178/authorguide#referencesformat

When submitting your revised manuscript, please carefully review the instructions that follow below. Failure to include requested items will delay the evaluation of your revision.

1) a .docx formatted version of the manuscript text (including legends for main figures, EV figures and tables). Please make sure that the changes are highlighted to be clearly visible.

2) individual production quality figure files as .eps, .tif, .jpg (one file per figure).

3) a .docx formatted letter INCLUDING the reviewers' reports and your detailed point-by-point responses to their comments. As part of the EMBO Press transparent editorial process, the point-by-point response is part of the Review Process File (RPF), which will be published alongside your paper. For more details on our Transparent Editorial Process, please visit our website: https://www.embopress.org/page/journal/14693178/authorguide#transparentprocess
You are able to opt out of this by letting the editorial office know (emboreports@embo.org). If you do opt out, the Review Process File link will point to the following statement: "No Review Process File is available with this article, as the authors have chosen not to make the review process public in this case."

4) a complete author checklist, which you can download from our author guidelines (<http://embor.embopress.org/authorguide>). Please insert information in the checklist that is also reflected in the manuscript. The completed author checklist will also be part of the RPF.

5) Please note that all corresponding authors are required to supply an ORCID ID for their name upon submission of a revised manuscript (<https://orcid.org/>). Please find instructions on how to link your ORCID ID to your account in our manuscript tracking system in our Author guidelines (<http://embor.embopress.org/authorguide>).

6) We replaced Supplementary Information with Expanded View (EV) Figures and Tables that are collapsible/expandable online. A maximum of 5 EV Figures can be typeset. EV Figures should be cited as 'Figure EV1, Figure EV2" etc... in the text and their respective legends should be included in the main text after the legends of regular figures.

- For the figures that you do NOT wish to display as Expanded View figures, they should be bundled together with their legends in a single PDF file called *Appendix*, which should start with a short Table of Content. Appendix figures should be referred to in the main text as: "Appendix Figure S1, Appendix Figure S2" etc. See detailed instructions regarding expanded view here: <http://embor.embopress.org/authorguide#expandedview>.

- Additional Tables/Datasets should be labeled and referred to as Table EV1, Dataset EV1, etc.

Legends have to be provided in a separate tab in case of .xls files. Alternatively, the legend can be supplied as a separate text file (README) and zipped together with the Table/Dataset file.

7) We would also encourage you to include the source data for figure panels that show essential data.

Numerical data should be provided as individual .xls or .csv files (including a tab describing the data). For blots or microscopy, uncropped images should be submitted (using a zip archive if multiple images need to be supplied for one panel). Additional information on source data and instruction on how to label the files are available <http://embor.embopress.org/authorguide#sourcedata>.

8) Our journal encourages inclusion of *data citations in the reference list* to directly cite datasets that were re-used and obtained from public databases. Data citations in the article text are distinct from normal bibliographical citations and should directly link to the database records from which the data can be accessed. In the main text, data citations are formatted as follows: "Data ref: Smith et al, 2001" or "Data ref: NCBI Sequence Read Archive PRJNA342805, 2017". In the Reference list, data citations must be labeled with "[DATASET]". A data reference must provide the database name, accession number/identifiers and a resolvable link to the landing page from which the data can be accessed at the end of the reference. Further instructions are available at <http://embor.embopress.org/authorguide#datacitation>.

9) Please make sure to include a Data Availability Section before submitting your revision - if it is not applicable, make a statement that no data were deposited in a public database. Primary datasets (and computer code, where appropriate) produced in this study need to be deposited in an appropriate public database (see <http://embor.embopress.org/authorguide#dataavailability>).

Please remember to provide a reviewer password if the datasets are not yet public.

The accession numbers and database should be listed in a formal "Data Availability " section (placed after Materials & Method) that follows the model below. Please note that the Data Availability Section is restricted to new primary data that are part of this study.

Data availability

The datasets (and computer code) produced in this study are available in the following databases:

- RNA-Seq data: Gene Expression Omnibus GSE46843 (https://www.ncbi.nlm.nih.gov/geo/query/acc.cgi?acc=GSE46843)
- [data type]: [name of the resource] [accession number/identifier/doi] ([URL or identifiers.org/DATABASE:ACCESSION])

*** Note - All links should resolve to a page where the data can be accessed. ***

10) Regarding data quantification, please ensure to specify the name of the statistical test used to generate error bars and P values, the number (n) of independent experiments underlying each data point (not replicate measures of one sample), and the test used to calculate p-values in each figure legend. Discussion of statistical methodology can be reported in the materials and methods section,

but figure legends should contain a basic description of n, P and the test applied.
Please note that error bars and statistical comparisons may only be applied to data obtained from at least three independent biological replicates.
Please also include scale bars in all microscopy images.

We would also welcome the submission of cover suggestions, or motifs to be used by our Graphics Illustrator in designing a cover.

I look forward to seeing a revised version of your manuscript when it is ready. Please let me know if you have questions or comments regarding the revision.

Yours sincerely,

Deniz Senyilmaz Tiebe

Deniz Senyilmaz Tiebe, PhD
Editor
EMBO Reports

Referee #1:

The manuscript addresses the highly controversial topic of the physiological role of respiratory supercomplexes. The authors were able to obtain a high resolution structural analysis of the yeast mitochondrial respiratory supercomplex. They identified key residues in the interaction interface and generated site-specific mutants that disrupt supercomplex formation but do not disturb the function of the individual respiratory complexes. By this elegant approach, they were able to obtain unprecedented insight in the functional role of respiratory supercomplexes. They demonstrate that the efficient diffusion of cytochrome c between complexes III and IV represents a crucial function of supercomplexes.

The data is of excellent quality and the manuscript is written in a clear and concise style. The paper will represent a milestone in understanding the structure-function organization of the mitochondrial respiratory chain.

Points to be addressed:

1. The title sounds quite general. In case space permits, it would be very good to include: diffusion of cytochrome c

2. The authors discuss shortly the various controversial models that have been proposed for the role of respiratory supercomplexes, yet due to space limitations they cannot explain the models for the general readership. I suggest to include an additional Table that presents the various models and their suggestions/conclusions and open questions/problems and includes the implications of this study.

Referee #2:

This is an interesting article about the possible mechanistic role of so-called supercomplex arrangement of the catalytic complexes of the respiratory chain. The study makes excellent use of the X-ray structures of the yeast supercomplex (III)2IV, i.e. between a dimer of the cytochrome bc1 complex and a monomer of cytochrome c oxidase. After identifying key residues for making the necessary contact to form the supercomplex, careful mutations of such residues are done to yield a new situation where apparently the complexes are still present in normal amounts but no longer form supercomplexes. Assays at different levels of organisation suggests that electron transfer from NADH to O2, or from succinate to O2 is reduced in the mutant suggesting that the supercomplex is important for assuring maximum efficiency electron transfer between complexes III and IV, via cyt c.
There is one problem with the spectroscopy data. Materials and Methods describes methods to assess the contents of cytochrome aa3, heme b, and heme c1 from the samples. However, no mention is given of determining the content of cyt c. The method of determining hemes b and c1 was designed for a pure bc1 complex, yet Table EV2 gives values of "heme c". It is nevertheless clear that cyt c is included based on the given heme contents, which should yield a content of heme c1 of only half the content of heme b. It is important to determine the cyt c content separately, not least to control that the cyt c concentration has not been diminished in the mutant, thus causing the reduced activity. Table EV2 gives the concentrations in uM, but there is no reference to the amount of protein in the different samples. One might of course infer that the content of heme c1 is half that measured for heme b, and subtract that from the results using the method described to yield a relative estimate of cyt c content. However, that is unlikely to yield the correct absolute amount of cyt c due to the methodology.
Finally, a more general question. Most mitochondria contain larger amounts of complex IV than complex III, the commonplace number is two-fold. In this work it seems to be about 1.5 fold. This means that with the (III)2IV stoichiometry of the supercomplex, there will be 2-3 times more "free" complex IV units in the membrane than supercomplex units. A comment on how this would be in accordance with the present conclusions would be interesting.

Referee #3:

Respiratory chain complexes play a pivotal role for the energy metabolism. In mitochondria from baker´s yeast, complex III and complex IV associate in supercomplexes. However, the function of the supercomplexes remains unclear. To analyze the role of the supercomplexes, Berndtsson and colleagues used yeast mutant strains with disrupted supercomplexes. Based on their structural model, the authors identified a binding site between Cor1 and Cox5a. Screening various Cor1 variants, they identified a mutant, termed Cor1**, in which the supercomplexes are largely dissociated, but the levels on the individual complexes remain comparable to wild-type mitochondria. Using this mutant strain and an elegant set of biochemical assays, the authors found that formation of respiratory chain supercomplexes promotes the transfer of electrons via cytochrome c and thereby promotes competitive fitness. Overall, the study is well written and the experimental data are of high quality. The conclusions are well-based on experimental findings. I have only minor recommendations for the revision.

The authors show that the respiratory supercomplexes are destabilized in the Cor1** mutants. However, in Figure 1A a small amount of respiratory chain supercomplexes is still detectable using the Cox1 antibody. The authors should adjust their conclusions accordingly (Page 4, first paragraph, last sentence). Alternatively, the authors may also perform pulldowns to verify that complex III and complex IV are dissociated.

The authors showed that addition of cytochrome c rescues NADH oxidation in mutant mitoplasts (Figure 4F). Based on this finding the authors conclude that supercomplex formation facilitates electron transfer from complex III to complex IV via cytochrome c. The conclusion is reasonable. Can the authors provide any experimental data to support this model in intact mitochondria or in cells? For instance, does overexpression of cytochrome c restore the competitive fitness of the mutant cells?

We would like to thank the three reviewers for their work with our manuscript, and the very positive evaluation of the work. The three referees raised a number of points that helped us to prepare an improved version. Please find below a point-by-point description of how we implemented their suggestions.

Referee #1:

The manuscript addresses the highly controversial topic of the physiological role of respiratory supercomplexes. The authors were able to obtain a high resolution structural analysis of the yeast mitochondrial respiratory supercomplex. They identified key residues in the interaction interface and generated site-specific mutants that disrupt supercomplex formation but do not disturb the function of the individual respiratory complexes. By this elegant approach, they were able to obtain unprecedented insight in the functional role of respiratory supercomplexes. They demonstrate that the efficient diffusion of cytochrome c between complexes III and IV represents a crucial function of supercomplexes.
The data is of excellent quality and the manuscript is written in a clear and concise style. The paper will represent a milestone in understanding the structure-function organization of the mitochondrial respiratory chain.
Points to be addressed:

1. The title sounds quite general. In case space permits, it would be very good to include: diffusion of cytochrome c

We thank the referee for this comment and changed the title to: *“Respiratory supercomplex formation enhances electron transport via cytochrome c diffusion”.*

2. The authors discuss shortly the various controversial models that have been proposed for the role of respiratory supercomplexes, yet due to space limitations they cannot explain the models for the general readership. I suggest to include an additional Table that presents the various models and their suggestions/conclusions and open questions/problems and includes the implications of this study.

We agree with the referee that the very brief explanation of the models regarding the role of respiratory supercomplexes might be too succinct, especially for the general readership. Since space limitations permitted an extension of the text, we decided to describe the models in more detail in the introduction.

Referee #2:

This is an interesting article about the possible mechanistic role of so-called supercomplex arrangement of the catalytic complexes of the respiratory chain. The study makes excellent use of the X-ray structures of the yeast supercomplex (III)2IV, i.e. between a dimer of the cytochrome bc1 complex and a monomer of cytochrome c oxidase. After identifying key residues for making the necessary contact to form the supercomplex, careful mutations of such residues are done to yield a new situation where apparently the complexes are still present in normal amounts but no longer form supercomplexes. Assays at different levels of organisation suggests that electron transfer from NADH to O2, or from succinate to O2 is reduced in the mutant suggesting that the supercomplex is important for assuring maximum efficiency electron transfer between complexes III and IV, via cyt c.

There is one problem with the spectroscopy data. Materials and Methods describes methods to assess the contents of cytochrome aa3, heme b, and heme c1 from the samples. However, no mention is given of determining the content of cyt c. The method of determining hemes b and c1 was designed for a pure bc1 complex, yet Table EV2 gives values of "heme c". It is nevertheless clear that cyt c is included based on the given heme contents, which should yield a content of heme c1 of only half the content of heme b. It is important to determine the cyt c content separately, not least to control that the cyt c concentration has not been diminished in the mutant, thus causing the reduced activity.
Table EV2 gives the concentrations in uM, but there is no reference to the amount of protein in the different samples. One might of course infer that the content of heme c1 is half that measured for heme b, and subtract that from the results using the method described to yield a relative estimate of cyt c content. However, that is unlikely to yield the correct absolute amount of cyt c due to the methodology.

We thank the referee for this critical comment. To clarify that the given heme *c* content includes both heme *c* and heme c_1, we now relabeled this quantification of *c*-type hemes to “heme cc_1” in both the table and the respective material and method sections. We agree that it is important to analyze the heme *c* content separately from heme c_1, thus we performed additional experiments. We generated mitoplasts via hypotonic treatment to release cytochrome *c* and separated it from mitoplasts by centrifugation. Performing spectroscopic analyses from the supernatant allowed us to quantify heme *c* individually and revealed that the Cor1** mutant had heme c levels comparable to wild type cells, which is in line with unchanged cytochrome *c* protein levels presented in Fig. 2 D. Hence, we can exclude that observed phenotypes for the Cor1** mutant are due to diminished heme *c* or reduced cytochrome *c* protein levels. We added a description of these phenotypes in the respective result section and updated the material and method section accordingly.
We apologize for the confusion regarding the given concentrations/missing amount of protein in the samples. For every sample, 200 µg of protein (or the corresponding volume of supernatant in case of heme *c* quantification) was used. This was only briefly stated in the material and method section. To present this information more clearly, we now also adapted the header of table EV2 accordingly.

Finally, a more general question. Most mitochondria contain larger amounts of complex IV than complex III, the commonplace number is two-fold. In this work it seems to be about 1.5 fold. This means that with the (III)2IV stoichiometry of the supercomplex, there will be 2-3 times more "free" complex IV units in the membrane than supercomplex units. A comment on how this would be in accordance with the present conclusions would be interesting.

In yeast mitochondria supercomplexes exist in two different stoichiometries, namely $CIII_2CIV$, and $CIII_2CIV_2$.Therefore most of the CIV and CIII exist in supercomplexes, which is also evident from the Western blot analysis presented in figure 2A. It is therefore highly likely that the organization of the respiratory chain in supercomplexes conveys a substantial advantage for electron transport. The minor fraction of free CIV is therefore expected not to play a major role for the efficiency of OXPHOS, but this will require further experiments to address properly.

Referee #3:

Respiratory chain complexes play a pivotal role for the energy metabolism. In mitochondria from baker´s yeast, complex III and complex IV associate in supercomplexes. However, the function of the supercomplexes remains unclear. To analyze the role of the supercomplexes, Berndtsson and colleagues used yeast mutant strains with disrupted supercomplexes. Based on their structural model, the authors identified a binding site between Cor1 and Cox5a. Screening various Cor1 variants, they identified a mutant, termed Cor1**, in which the supercomplexes are largely dissociated, but the levels on the individual complexes remain comparable to wild-type mitochondria. Using this mutant strain and an elegant set of biochemical assays, the authors found that formation of respiratory chain supercomplexes promotes the transfer of electrons via cytochrome c and thereby promotes competitive fitness. Overall, the study is well written and the experimental data are of high quality. The conclusions are well-based on experimental findings. I have only minor recommendations for the revision.

The authors show that the respiratory supercomplexes are destabilized in the Cor1** mutants. However, in Figure 1A a small amount of respiratory chain supercomplexes is still detectable using the Cox1 antibody. The authors should adjust their conclusions accordingly (Page 4, first paragraph, last sentence). Alternatively, the authors may also perform pulldowns to verify that complex III and complex IV are dissociated.

We thank the referee for this critical comment. As no corresponding signal for CIII in immunoblots probed with the anti Cor1 antibody can be detected, it can be assumed that supercomplexes are entirely destabilized, which also correlates with presented Coomassie stained gels. Thus, we rephrased the mentioned paragraph/sentence as follows:

Two of these mutants, namely Cor1$^{N63A,\ N187A,\ D192A}$ (hereafter Cor1*), and Cor1$^{N63A,\ N187A,\ D192A,\ Y65A,\ V189A,\ L238A,\ K240A}$ (Cor1**), lacked higher molecular weight complexes containing both CIII and CIV, thus revealing complete SC disruption (Fig. 1 D).

The authors showed that addition of cytochrome c rescues NADH oxidation in mutant mitoplasts (Figure 4F). Based on this finding the authors conclude that supercomplex formation facilitates electron transfer from complex III to complex IV via cytochrome c. The conclusion is reasonable. Can the authors provide any experimental data to support this model in intact mitochondria or in cells? For instance, does overexpression of cytochrome c restore the competitive fitness of the mutant cells?

We thank the referee for this excellent suggestion that we have addressed experimentally. We overexpressed cytochrome *c* to monitor a potential restoration of NADH driven respiration in isolated mitochondria and competitive fitness of the Cor1** mutant. Despite the fact that cytochrome c is described to be a pro-apoptotic protein in both yeast and mammals, our analysis revealed that no increase in cell death nor growth retardation was caused by increased levels of cytochrome *c* within the first 24 hours after inoculation. Importantly increased levels of cytochrome *c* corrected the decreased NADH driven respiration in supercomplex-lacking mitochondria. Moreover, overexpression of cytochrome *c* restored competitive fitness of the Cor1** mutant. These results now strengthen our hypothesis that

supercomplexes determine competitive fitness via enhancing the efficiency of electron transfer via cytochrome *c*.

1st Revision - Editorial Decision 31st Aug 2020

Dear Martin,

Thank you for submitting the revised version of your manuscript. It has now been seen by one of the original referees.

As you can see, the referee finds that the study is significantly improved during revision and recommends publication. Before I can accept the manuscript, I need you to address some minor points below:

• We noticed that the reference format should be corrected as follows: where there are more than 10 authors on a paper, 10 will be listed, followed by 'et al.'. Please see https://www.embopress.org/page/journal/14693178/authorguide#referencesformat for more details.
• Please make the data mentioned in he Data Availability section publicly available (CIII2/CIV (EMD-10847 and 6YMX) and CIV model (EMD-10848 and 6YMY)).

Thank you again for giving us to consider your manuscript for EMBO Reports, I look forward to your minor revision.

Kind regards,

Deniz

--
Deniz Senyilmaz Tiebe, PhD
Editor
EMBO Reports

Referee #3:

The authors fully addressed all my concerns in the revised version. The new data nicley support the conclusions drawn by the authors. The manuscript provides a highy interesting findings for the role respiratory chain supercomplexes. I strongly recommend publication of this manuscript in EMBO rep..

The authors have addressed all minor editorial issues.

2nd Revision - Editorial Decision 10th Sep 2020

Dear Martin,

Thank you for submitting your revised manuscript. I have now looked at everything and all is fine. Therefore I am very pleased to accept your manuscript for publication in EMBO Reports.

Congratulations on a nice study!

Kind regards,

Deniz

--
Deniz Senyilmaz Tiebe, PhD
Editor
EMBO Reports

At the end of this email I include important information about how to proceed. Please ensure that you take the time to read the information and complete and return the necessary forms to allow us to publish your manuscript as quickly as possible.

As part of the EMBO publication's Transparent Editorial Process, EMBO reports publishes online a Review Process File to accompany accepted manuscripts. As you are aware, this File will be published in conjunction with your paper and will include the referee reports, your point-by-point response and all pertinent correspondence relating to the manuscript.

If you do NOT want this File to be published, please inform the editorial office within 2 days, if you have not done so already, otherwise the File will be published by default [contact: emboreports@embo.org]. If you do opt out, the Review Process File link will point to the following statement: "No Review Process File is available with this article, as the authors have chosen not to make the review process public in this case."

Should you be planning a Press Release on your article, please get in contact with emboreports@wiley.com as early as possible, in order to coordinate publication and release dates.

Thank you again for your contribution to EMBO reports and congratulations on a successful publication. Please consider us again in the future for your most exciting work.

****

THINGS TO DO NOW:

You will receive proofs by e-mail approximately 2-3 weeks after all relevant files have been sent to our Production Office; you should return your corrections within 2 days of receiving the proofs.

Please inform us if there is likely to be any difficulty in reaching you at the above address at that time. Failure to meet our deadlines may result in a delay of publication, or publication without your corrections.

All further communications concerning your paper should quote reference number EMBOR-2020-51015V3 and be addressed to emboreports@wiley.com.

Should you be planning a Press Release on your article, please get in contact with emboreports@wiley.com as early as possible, in order to coordinate publication and release dates.

EMBO PRESS

YOU MUST COMPLETE ALL CELLS WITH A PINK BACKGROUND ↓

PLEASE NOTE THAT THIS CHECKLIST WILL BE PUBLISHED ALONGSIDE YOUR PAPER

Corresponding Author Name: Martin Ott	
Journal Submitted to: EMBO Reports	
Manuscript Number: EMBOR-2020-51015V1	

Reporting Checklist For Life Sciences Articles (Rev. June 2017)

This checklist is used to ensure good reporting standards and to improve the reproducibility of published results. These guidelines are consistent with the Principles and Guidelines for Reporting Preclinical Research issued by the NIH in 2014. Please follow the journal's authorship guidelines in preparing your manuscript.

A- Figures
1. Data
The data shown in figures should satisfy the following conditions:

- the data were obtained and processed according to the field's best practice and are presented to reflect the results of the experiments in an accurate and unbiased manner.
- figure panels include only data points, measurements or observations that can be compared to each other in a scientifically meaningful way.
- graphs include clearly labeled error bars for independent experiments and sample sizes. Unless justified, error bars should not be shown for technical replicates.
- if n< 5, the individual data points from each experiment should be plotted and any statistical test employed should be justified
- Source Data should be included to report the data underlying graphs. Please follow the guidelines set out in the author ship guidelines on Data Presentation.

2. Captions
Each figure caption should contain the following information, for each panel where they are relevant:

- a specification of the experimental system investigated (eg cell line, species name).
- the assay(s) and method(s) used to carry out the reported observations and measurements
- an explicit mention of the biological and chemical entity(ies) that are being measured.
- an explicit mention of the biological and chemical entity(ies) that are altered/varied/perturbed in a controlled manner.

- the exact sample size (n) for each experimental group/condition, given as a number, not a range;
- a description of the sample collection allowing the reader to understand whether the samples represent technical or biological replicates (including how many animals, litters, cultures, etc.).
- a statement of how many times the experiment shown was independently replicated in the laboratory.
- definitions of statistical methods and measures:
  - common tests, such as t-test (please specify whether paired vs. unpaired), simple χ2 tests, Wilcoxon and Mann-Whitney tests, can be unambiguously identified by name only, but more complex techniques should be described in the methods section;
  - are tests one-sided or two-sided?
  - are there adjustments for multiple comparisons?
  - exact statistical test results, e.g., P values = x but not P values < x;
  - definition of 'center values' as median or average;
  - definition of error bars as s.d. or s.e.m.

Any descriptions too long for the figure legend should be included in the methods section and/or with the source data.

In the pink boxes below, please ensure that the answers to the following questions are reported in the manuscript itself. Every question should be answered. If the question is not relevant to your research, please write NA (non applicable). We encourage you to include a specific subsection in the methods section for statistics, reagents, animal models and human subjects.

USEFUL LINKS FOR COMPLETING THIS FORM

http://www.antibodypedia.com
http://1degreebio.org
http://www.equator-network.org/reporting-guidelines/improving-bioscience-research-repor
http://grants.nih.gov/grants/olaw/olaw.htm
http://www.mrc.ac.uk/Ourresearch/Ethicsresearchguidance/Useofanimals/index.htm
http://ClinicalTrials.gov
http://www.consort-statement.org
http://www.consort-statement.org/checklists/view/32-consort/66-title
http://www.equator-network.org/reporting-guidelines/reporting-recommendations-for-tumo
http://datadryad.org
http://figshare.com
http://www.ncbi.nlm.nih.gov/gap
http://www.ebi.ac.uk/ega
http://biomodels.net/
http://biomodels.net/miriam/
http://jjj.biochem.sun.ac.za
http://oba.od.nih.gov/biosecurity/biosecurity_documents.html
http://www.selectagents.gov/

B- Statistics and general methods

Please fill out these boxes ↓ (Do not worry if you cannot see all your text once you press return)

1.a. How was the sample size chosen to ensure adequate power to detect a pre-specified effect size?	At least 3 different biological replicates (3 independent clones per yeast strain or 3 independent mitochondrial preparations) were analysed per experiment. No sample size calculation was performed; samples sizes were chosen according to empirical values that are standard in the field, providing sufficient power to detect physiologically relevant changes.
1.b. For animal studies, include a statement about sample size estimate even if no statistical methods were used.	NA
2. Describe inclusion/exclusion criteria if samples or animals were excluded from the analysis. Were the criteria pre-established?	Cor1* mutants were excluded from further analysis after experiments presented in Figure 2, as this mutant displayed altered accumulation of CIII subunits. Since Cor1** also disrupted supercomplexes and did not impair protein levels of CIII or CIV subunits, we analysed this strain to selectively evaluate physiological consequences of supercomplex disruption. Cardiolipin synthase knockout strains were not used for experiments conducted after those shown in Figure 2, since Cor1** was sufficient to disrupt supercomplex formation and cardiolipin synthase knockouts per se had no effect on supercomplex formation. Respective data leading to these decisions are presented in Figure 2, and are described and discussed in the manuscript. Outliers were defined as data points outside the 2.2-fold interquartile range (IQR) and are highlighted in turquoise. Upon presence of outliers, alternative non-parametric tests were performed (described in detail in Table EV6). Of note, outliers were not excluded from the analysis.
3. Were any steps taken to minimize the effects of subjective bias when allocating animals/samples to treatment (e.g. randomization procedure)? If yes, please describe.	No randomization was performed. Covariates were excluded by using isogenic yeast strains, independent clones to exclude clonogenic variations, simultaneous inoculation of all strains within an experiment to equal optical density, using the same batch of media. In competitive fitness analyses, different selection markers had to be used for wild type cells and Cor1 mutants. Hence, control experiments were performed with switched selection markers and results are presented in the manuscript.
For animal studies, include a statement about randomization even if no randomization was used.	NA
4.a. Were any steps taken to minimize the effects of subjective bias during group allocation or/and when assessing results (e.g. blinding of the investigator)? If yes please describe.	No blinding was performed in this study, as measurements and/or analyses did not allow subjective evaluation of the data.
4.b. For animal studies, include a statement about blinding even if no blinding was done	NA
5. For every figure, are statistical tests justified as appropriate?	The number of n given for each experiment represents biological replicates. Thereby, experiments were performed with several clones (at least 3 per genotype) to exclude clonogenic variations or with mitochondria obtained from individual preparations (at least 3 per strain). A detailed description of statistical analysis is given in the method section.
Do the data meet the assumptions of the tests (e.g., normal distribution)? Describe any methods used to assess it.	Assumptions for statistical tests were carefully evaluated as described in the method section. In brief, we applied Shapiro-Wilk's test to evaluate normal distribution, Levene's test to test for homogeneity of variances and outliers were detected via 2.2-fold interquartile range labelling rule. Upon violation of assumptions, respective non-parametic test were performed or adequate correction methods (e.g. Welch correction upon significantly different variances) were applied. A detailed description of the procedure upon violation of respective assumptions is presented in Table EV6.
Is there an estimate of variation within each group of data?	Standard error of the mean (SEM) was calculated for each group of all datasets analysed

Is the variance similar between the groups that are being statistically compared?	Homogeneity of variances were evaluated via Levene's test, and results and respective procedures upon violation are presented in Table EV6.

C- Reagents

6. To show that antibodies were profiled for use in the system under study (assay and species), provide a citation, catalog number and/or clone number, supplementary information or reference to an antibody validation profile. e.g., Antibodypedia (see link list at top right), 1DegreeBio (see link list at top right).	Anti-Cox1 Abcam Cat#ab110270; Anti-Cox5 (Liu and Barrientos, 2013); Anti-Cox12 Gift from Dr. J. Brix; Anti-Cox13 Gift from Dr. J. Brix; Anti-Cytb Gift from Dr. A. Tzagoloff; Anti-Rip1 Gift from Dr. R. Stuart; Anti-Cor1 Gift from Dr. A. Tzagoloff; Anti-Cytc (Barrientos et al., 2003); Anti-Rcf1 Gift from Dr. R. Stuart; Anti-Cmc2 (Horn et al., 2008); Anti-Porin Abcam Cat#ab110326; Anti-Tom70 (Hildenbeutel et al., 2014); Anti-Cor1 (Hildenbeutel et al., 2014); Anti-Cox2 (Hildenbeutel et al., 2014); Anti-Qcr7 (Gruschke et al., 2012), Anti-tubulin Abcam Cat#ab184970<br><br>Barrientos, A., Pierre, D., Lee, J., and Tzagoloff, A. (2003). Cytochrome oxidase assembly does not require catalytically active cytochrome c. J. Biol. Chem. 278, 8881–8887.<br>Gruschke, S., Römpler, K., Hildenbeutel, M., Kehrein, K., Kühl, I., Bonnefoy, N., and Ott, M. (2012). The Cbp3-Cbp6 complex coordinates cytochrome b synthesis with bc1 complex assembly in yeast mitochondria. J. Cell Biol. 199, 137–150.<br>Hildenbeutel, M., Hegg, E.L., Stephan, K., Gruschke, S., Meunier, B., and Ott, M. (2014). Assembly factors monitor sequential hemylation of cytochrome b to regulate mitochondrial translation. J. Cell Biol. 205, 511–524.<br>Horn, D., Fontanesi, F., and Barrientos, A. (2008). Exploring protein-protein interactions involving newly synthesized mitochondrial dna-encoded proteins. Methods Mol. Biol. 457, 125–139.<br>Liu, J., and Barrientos, A. (2013). Transcriptional regulation of yeast oxidative phosphorylation
7. Identify the source of cell lines and report if they were recently authenticated (e.g., by STR profiling) and tested for mycoplasma contamination.	All yeast strains in this study were isogenic to the intronless W303 strain MRSI0, kindly provided by A. Tzagoloff (Columbia University, New York, NY).

* for all hyperlinks, please see the table at the top right of the document

D- Animal Models

8. Report species, strain, gender, age of animals and genetic modification status where applicable. Please detail housing and husbandry conditions and the source of animals.	NA
9. For experiments involving live vertebrates, include a statement of compliance with ethical regulations and identify the committee(s) approving the experiments.	NA
10. We recommend consulting the ARRIVE guidelines (see link list at top right) (PLoS Biol. 8(6), e1000412, 2010) to ensure that other relevant aspects of animal studies are adequately reported. See author guidelines, under 'Reporting Guidelines'. See also: NIH (see link list at top right) and MRC (see link list at top right) recommendations. Please confirm compliance.	NA

E- Human Subjects

11. Identify the committee(s) approving the study protocol.	NA
12. Include a statement confirming that informed consent was obtained from all subjects and that the experiments conformed to the principles set out in the WMA Declaration of Helsinki and the Department of Health and Human Services Belmont Report.	NA
13. For publication of patient photos, include a statement confirming that consent to publish was obtained.	NA
14. Report any restrictions on the availability (and/or on the use) of human data or samples.	NA
15. Report the clinical trial registration number (at ClinicalTrials.gov or equivalent), where applicable.	NA
16. For phase II and III randomized controlled trials, please refer to the CONSORT flow diagram (see link list at top right) and submit the CONSORT checklist (see link list at top right) with your submission. See author guidelines, under 'Reporting Guidelines'. Please confirm you have submitted this list.	NA
17. For tumor marker prognostic studies, we recommend that you follow the REMARK reporting guidelines (see link list at top right). See author guidelines, under 'Reporting Guidelines'. Please confirm you have followed these guidelines.	NA

F- Data Accessibility

18: Provide a "Data Availability" section at the end of the Materials & Methods, listing the accession codes for data generated in this study and deposited in a public database (e.g. RNA-Seq data: Gene Expression Omnibus GSE39462, Proteomics data: PRIDE PXD000208 etc.) Please refer to our author guidelines for 'Data Deposition'.<br><br>Data deposition in a public repository is mandatory for:<br>a. Protein, DNA and RNA sequences<br>b. Macromolecular structures<br>c. Crystallographic data for small molecules<br>d. Functional genomics data<br>e. Proteomics and molecular interactions	Cryo-EM maps and atomic coordinates have been deposited at the Electron Microscopy Data Bank (EMD) and the Protein Data Bank (PDB) with the accession codes for the CIII2/CIV (EMD-10847 and 6YMX) and CIV model (EMD-10848 and 6YMY).
19. Deposition is strongly recommended for any datasets that are central and integral to the study; please consider the journal's data policy. If no structured public repository exists for a given data type, we encourage the provision of datasets in the manuscript as a Supplementary Document (see author guidelines under 'Expanded View' or in unstructured repositories such as Dryad (see link list at top right) or Figshare (see link list at top right).	
20. Access to human clinical and genomic datasets should be provided with as few restrictions as possible while respecting ethical obligations to the patients and relevant medical and legal issues. If practically possible and compatible with the individual consent agreement used in the study, such data should be deposited in one of the major public access-controlled repositories such as dbGAP (see link list at top right) or EGA (see link list at top right).	NA
21. Computational models that are central and integral to a study should be shared without restrictions and provided in a machine-readable form. The relevant accession numbers or links should be provided. When possible, standardized format (SBML, CellML) should be used instead of scripts (e.g. MATLAB). Authors are strongly encouraged to follow the MIRIAM guidelines (see link list at top right) and deposit their model in a public database such as Biomodels (see link list at top right) or JWS Online (see link list at top right). If computer source code is provided with the paper, it should be deposited in a public repository or included in supplementary information.	NA

G- Dual use research of concern

22. Could your study fall under dual use research restrictions? Please check biosecurity documents (see link list at top right) and list of select agents and toxins (APHIS/CDC) (see link list at top right). According to our biosecurity guidelines, provide a statement only if it could.	
